# Transcriptomics Investigation into the Mechanisms of Self-Incompatibility between Pin and Thrum Morphs of *Primula maximowiczii*

**DOI:** 10.3390/ijms19071840

**Published:** 2018-06-22

**Authors:** Wanpei Lu, Xiaomeng Bian, Weiru Yang, Tangren Cheng, Jia Wang, Qixiang Zhang, Huitang Pan

**Affiliations:** 1 Beijing Key Laboratory of Ornamental Plants Germplasm Innovation & Molecular Breeding, National Engineering Research Center for Floriculture, Beijing Laboratory of Urban and Rural Ecological Environment, Key Laboratory of Genetics and Breeding in Forest Trees and Ornamental Plants of Ministry of Education and College of Landscape Architecture, Beijing Forestry University, Beijing 100083, China; wanplu@163.com (W.L.); 13269895583@163.com (X.B.); yweiru@163.com (W.Y.); chengtangren@163.com (T.C.); wangjia8248@163.com (J.W.); zqxbjfu@126.com (Q.Z.); 2 Development Center of Science and Technology, Ministry of Agriculture and Rural Affairs, Beijing 100083, China

**Keywords:** *Primula maximowiczii*, distyly, heteromorphic self-incompatibility, differentially expressed genes, environmental adaptation

## Abstract

Heteromorphic self-incompatibility (SI) is an important system for preventing inbreeding in the genus *Primula*. However, investigations into the molecular mechanisms of *Primula* SI are lacking. To explore the mechanisms of SI in *Primula maximowiczii*, the pollen germination and fruiting rates of self- and cross-pollinations between pin and thrum morphs were investigated, and transcriptomics analyses of the pistils after pollination were performed to assess gene expression patterns in pin and thrum SI. The results indicated that *P. maximowiczii* exhibits strong SI and that the mechanisms of pollen tube inhibition differ between pin and thrum morphs. While self-pollen tubes of the pin morph were able to occasionally, though rarely, enter the style, those of the thrum morph were never observed to enter the style. The transcriptomics analysis of the pistils revealed 1311 and 1048 differentially expressed genes (DEGs) that were identified by comparing pin self-pollination (PS) vs. pin cross-pollination (PT) and thrum self-pollination (TS) vs. thrum cross-pollination (TP). Notably, about 90% of these DEGs exhibited different expression patterns in the two comparisons. Moreover, pin and thrum DEGs were associated with different Gene Ontology (GO) categories and Kyoto Encyclopedia of Genes and Genomes (KEGG) pathways following enrichment analyses. Based on our results, the molecular mechanisms underlying the pin and thrum SI in *P. maximowiczii* appear to be distinct. Furthermore, the genes involved in the SI processes are commonly associated with carbohydrate metabolism and environmental adaptation. These results provide new insight into the molecular mechanisms of *Primula* SI.

## 1. Introduction

Self-incompatibility (SI) is a common mechanism that promotes outcrossing in angiosperms. Based on floral morphology, SI can be classified as homomorphic or heteromorphic. Homomorphic SI systems include the gametophytic SI (GSI) and sporophytic SI (SSI) systems, and plants with homomorphic SI systems have no distinct floral morphologies [1]. Heteromorphic SI is usually considered sporophytic and is present in distylous and tristylous populations that possess long and short styles [2].

Heteromorphic SI has been reported in at least 28 families scattered throughout the angiosperm lineage [3]. In the Primulaceae family, floral morphs with long styles are termed pin morphs and those with short styles are termed thrum morphs. Dimorphic heteromorphic SI has been observed in 91% of *Primula* species, and this species is often considered to be a model system for heterostyly [4], with the two types of flowers varying in style length, anther position, and pollen size [5]. Classical theories proposed that heteromorphic SI systems are controlled by the S locus, with the thrum morph being heterozygous (*S*/*s*) and the pin being homozygous recessive (*s*/*s*) [6,7,8,9]. In contrast, Li et al. proposed that the S locus is actually hemizygous, not heterozygous, in thrum morphs [10]. Further studies indicated that three genes are present at the S locus as a co-adapted linkage group: *G* (style length and incompatibility), *P* (pollen size and incompatibility), and *A* (anther position) [2]. The first molecular marker for the *Primula* S locus was identified in *Primula vulgaris* [11], and two S locus-linked genes of *Primula* were identified using differential display technology [12]. The *Primula CYP734A50* gene was identified at the S locus and is expressed specifically in thrum styles [13], while the *GLOT*, *CYPT*, *PUMT*, *KFBT*, and *CCMT* genes were shown to be present at the S locus only in the thrum morph [10]. Moreover, *PvGLO2* was shown to be adjacent to *CYP734A50* which lies within the S locus [14]. However, the key genes that regulate heteromorphic SI in *Primula* remain unknown.

As mentioned above, heteromorphic SI is generally regarded as sporophytic. However, Shivanna et al. found that the thrum and pin morphs exhibited different self-pollen tube inhibition behaviors, with thrum self-pollen tube inhibition occurring on the stigma and pin self-pollen tube inhibition occurring frequently in the style [15]. Similarly, in the SI system of *P. obconica*, the thrum morph incompatibility barrier appears to be present on or in the stigma, while in the pin morph, the site of self-pollen tube inhibition could be the stigma or the style [16]. In two distylous *Turnera* species, obvious ultrastructural differences were observed between the pin and thrum self-pollen tubes [17]. Lewis and Jones proposed that the SI systems of thrum morphs may be sporophytic and those of pin morphs may be both sporophytic and gametophytic [2]. Thus, the SI mechanisms of pin and thrum morphs may be different.

Some researchers have proposed that SI systems originated from pathogen defense mechanisms [18,19,20,21]. The female S determinant of SSI in *Brassica* is the S-locus receptor kinase (SRK), a member of a large gene family that controls host–pathogen defense [22,23]. Similarly, the female S determinants of many GSI systems are S-locus ribonucleases (S-RNases), which are also involved in pathogen defense responses [24]. Some symptoms of programmed cell death (PCD), e.g., swollen mitochondria and nuclear DNA degradation, were observed in the self-pollen tubes of pear after incompatible pollination [25]. Similarly, PCD was observed in self-pollen tubes after interactions between the female and male S determinants in the *Papaver* SI system [26]. These findings demonstrate the close association between pathogen defense mechanisms and SI systems.

In recent years, most studies of the *Primula* SI system have focused on differences in pollination behaviors between morphs, such as the roles of bumblebee nectar robbers and syrphid flies in *P. secundiflora* pollination, the strength of SI and distylous syndrome in *P. veris*, the effect of anther–stigma distance on stigma pollen grains in two *Primula* species, and the relationship between flower display and SI in *P. tibetica* [27,28,29,30]. However, little is known about the biological events that occur during the process of *Primula* SI. In addition, while extensive molecular studies on *Primula* have been carried out, such as genomics analyses of *P. veris* and *P. vulgaris* [10,31], the regulatory factors involved in heteromorphic SI have not been identified. In the current study, we used *P. maximowiczii*, a perennial species native to Northern China, to investigate the pollen germination time, pollen tube elongation, and fruiting rates of self- and cross-pollinations of pin and thrum morphs, and we collected the pistils after four types of pollination to perform a transcriptomics analysis. Our results provide the first transcriptomics analysis of the heteromorphic SI of *Primula* and offer new data for future genetic and genomic studies on *Primula* SI.

## 2. Results

### 2.1. Heteromorphic SI of Distylous P. maximowiczii

**Figure 1:**
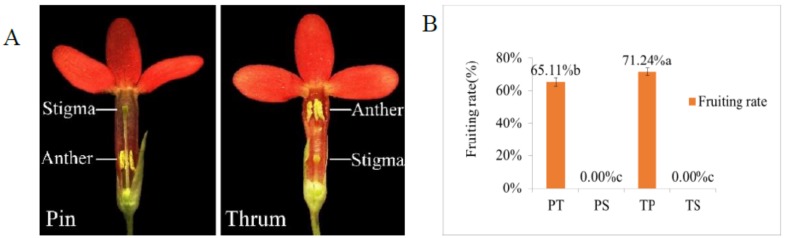
*P. maximowiczii* floral phenotypes and fruiting rates of different pollinations. (**A**) Pin flower with long style and thrum flower with short style; (**B**) Fruiting rates of different pollination treatments.

**Figure 2:**
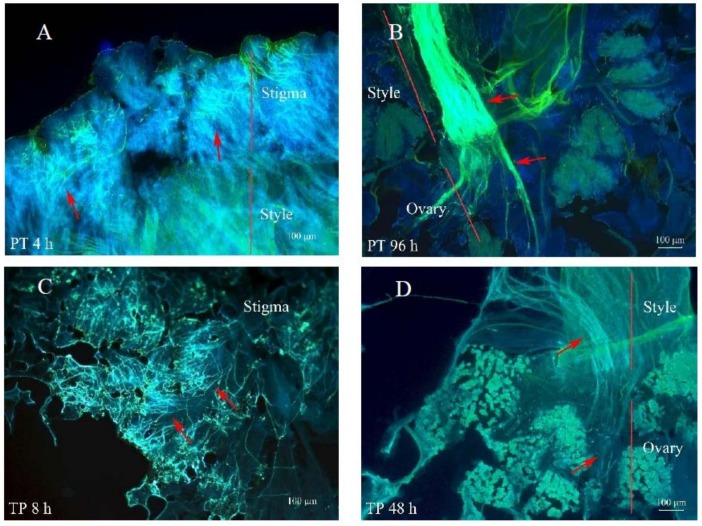

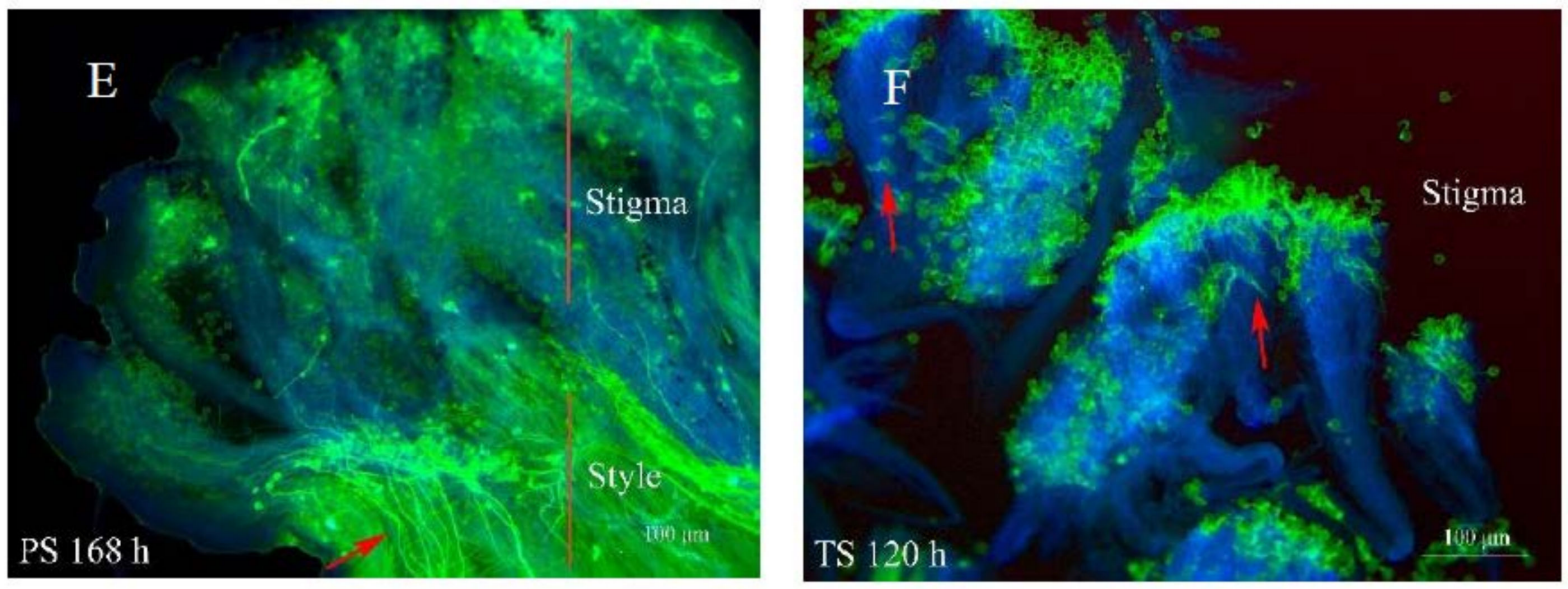
*P. maximowiczii* pollen germination and pollen tube growth after different pollination treatments. (**A**) In the pin cross-pollination (PT), pollen grains germinated and pollen tubes grew into the stigma at 4 h after pollination; (**B**) In the PT cross, pollen tubes entered the ovary at 96 h after pollination; (**C**) In the thrum cross-pollination (TP), pollen grains germinated and pollen tubes grew into the stigma at 8 h after pollination; (**D**) In the TP cross, pollen tubes entered the ovary at 48 h after pollination; (**E**) In the pin self-pollination (PS), a few pollen tubes entered the style at 168 h after pollination; (**F**) In the thrum self-pollination (TS), pollen grains germinated on the stigma at 120 h after pollination. Red arrows indicate pollen tubes. Red lines indicate various labeled sites, such as the stigma, style, and ovary.

*P. maximowiczii* is a distylous species with obvious reciprocal herkogamy in pin and thrum morphs (Figure 1A). We found that the fruiting rates of pin and thrum cross-pollinations (PT and TP) were 65.11% and 71.24%, respectively, while those of self-pollinations (PS and TS) were zero (Figure 1B). In PT pollinations, pollen grains started to germinate at 4 h, and the pollen tubes entered the ovary at 96 h after pollination (Figure 2A,B). In TP pollinations, pollen grains germinated and the pollen tubes entered the thrum stigma at 8 h, and then the pollen tubes grew into the ovary at 48 h after pollination (Figure 2C,D). In PS self-pollinations, the germination of pollen grains was only rarely observed, except at 168 h and 192 h, and the pollen tubes entered the pin styles in only a few cases (Figure 2E). In contrast, in TS self-pollinations, pollen grains usually germinated on the stigmas, but no pollen tubes grew into the thrum styles (Figure 2F; see Supplementary Figures S1–S4 for photographs of other observations at different time points after pollination). These results indicate that the compatible pollen of PT and TP crosses started to geminate at 4 and 8 h after pollination, respectively, while the pollen tubes of PS self-crosses rarely entered the style and those of TS self-crosses never entered the style.

### 2.2. De Novo RNA-seq Assembly and Annotation of Unigenes

In order to explore the molecular mechanism underlying the heteromorphic SI of *P. maximowiczii*, we performed de novo transcriptomics sequencing analyses of the pistils of PT, PS, TP, and TS crosses. In total, 57,893,634,000 bp of sequence data was generated in this project. After data cleaning, we obtained an average of 53,605,217 clean reads for each sample with average guanine and cytosine (GC) contents of the reads of 42.99% (Table S1). After assembling and clustering the high-quality reads, 99,754 unigenes were identified, with an N50 of 1907 nt (Table S2). For the functional annotation analysis, more than 61% (61,552) of the total unigenes were annotated using various databases, including the Non-Redundant Protein Sequence (NR) (59,798), Nucleotide Sequence Database (NT) (48,174), Swiss-Prot (39,419), Kyoto Encyclopedia of Genes and Genomes (KEGG) (36,120), Cluster of Orthologous Groups of proteins (COG) (25,123), and Gene Ontology (GO) (45,456) databases (Table 1). There were 59,014 unigenes with a coding sequence (CDS) predicted from the protein databases, and 2741 unigenes with a CDS predicted by ESTScan. The total number of unigenes with a predicted CDS was 61,755, representing 61.91% of all unigenes.

### 2.3. Differentially Expressed Genes (DEGs) in Pin and Thrum SI

To investigate the expression of genes during SI, we used the metric fragments per kilobase of transcript per million mapped reads (FPKM) and assessed the expression level of each unigene in the pin and thrum pistils following self-incompatible and compatible pollination (PS vs. PT and TS vs. TP). We identified 1311 and 1048 DEGs in the PS vs. PT and TS vs. TP comparisons, respectively, to give a total of 2135 genes (Tables S3 and S4). In addition, 3400, 3509, 3559, and 3185 DEGs were identified in the TP vs. PT, TS vs. PT, TP vs. PS, and TS vs. PS comparisons, respectively. Thus, the number of DEGs observed in comparisons between the pin and thrum morphs were clearly greater than those observed in intra-morph comparisons. However, because the aim of the study was to investigate the heteromorphic SI system of *P. maximowiczii*, we focused on the DEGs derived from the PS vs. PT and TS vs. TP comparisons.

**Figure 3:**
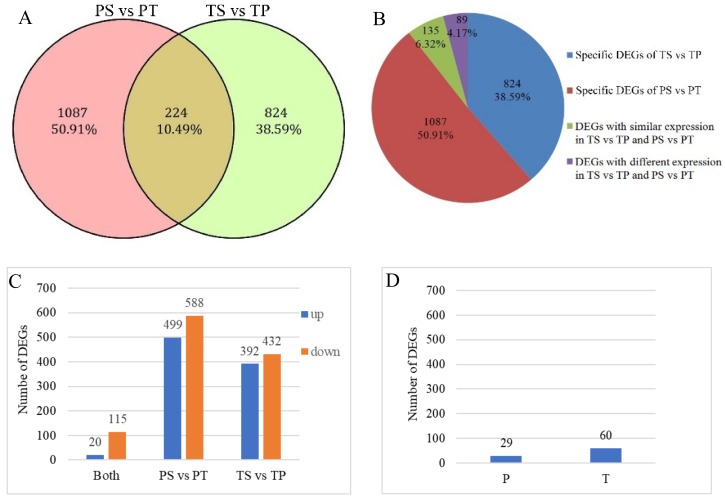

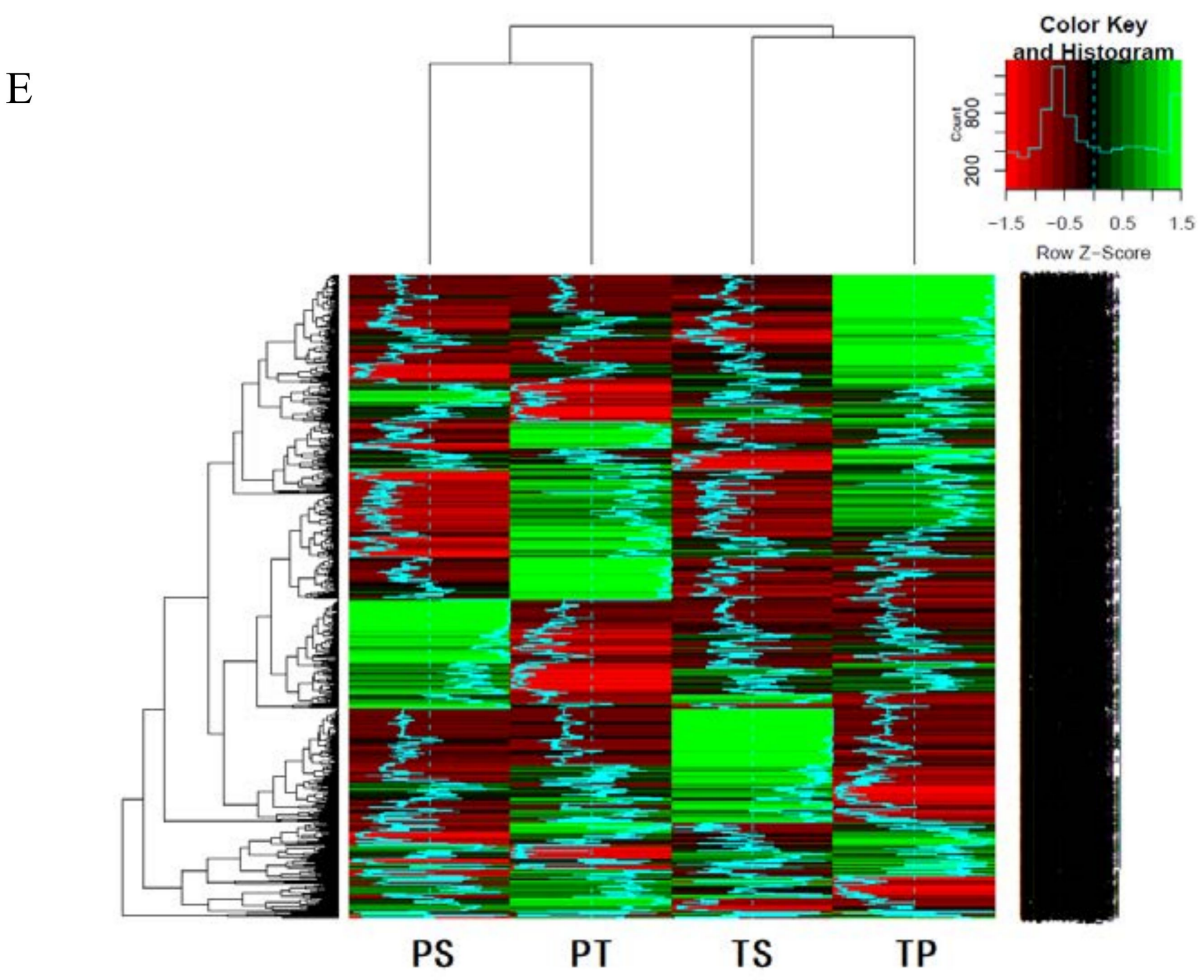
Venn diagram of the number of Differentially Expressed Genes (DEGs) and heat map of the cluster analysis of total DEGs. (**A**) Venn diagram of the number of DEGs between pin and thrum comparisons; (**B**) Distribution of DEGs in different groups, including those specific to the TS vs. TP and PS vs. PT comparisons, those with similar expression patterns in the TS vs. TP and PS vs. PT comparisons, and those with different expression patterns in the TS vs. TP and PS vs. PT comparisons; (**C**) Number of DEGs upregulated and downregulated in the pin and thrum comparisons. “Both” indicates DEGs with similar expression patterns in the TS vs. TP and PS vs. PT comparisons; “PS vs. PT” indicates DEGs specific to the PS vs. PT comparison; and “TS vs. TP” indicates DEGs specific to the TS vs. TP comparison; (**D**) Number of DEGs with different expression patterns in the TS vs. TP and PS vs. PT comparisons. P indicates DEGs upregulated in the PS vs. PT comparison and downregulated in the TS vs. TP comparison; and T indicates DEGs upregulated in the TS vs. TP comparison and downregulated in the PS vs. PT comparison; (**E**) Heat map of the cluster analysis of all 2135 DEGs from the PS vs. PT and TS vs. TP comparisons; each column contains 2135 unigenes.

A total of 224 of the 2135 DEGs were differentially expressed in both the PS vs. PT and TS vs. TP comparisons, with 6.32% of all DEGs having similar expression patterns in the PS vs. PT and TS vs. TP comparisons and 4.17% having different expression patterns. In contrast, 1087 and 824 DEGs were specific to the PS vs. PT and TS vs. TP comparisons, respectively (Figure 3A,B), accounting for 89.51% of the 2135 DEGs. Thus, the total number of DEGs with different expression patterns between the two comparisons accounted for 93.68% (Figure 3B). Among the DEGs with similar expression patterns in the TS vs. TP and PS vs. PT comparisons, 20 DEGs were upregulated and 115 DEGs were downregulated (Figure 3C, Table S5). Meanwhile, 499 upregulated and 588 downregulated DEGs were specific to the PS vs. PT comparison, and 392 upregulated and 432 downregulated DEGs were specific to the TS vs. TP comparison (Figure 3C, Tables S3 and S4). Among the DEGs with different expression patterns in the TS vs. TP and PS vs. PT comparisons, 29 DEGs were upregulated in the PS vs. PT comparison and downregulated in the TS vs. TP comparison, and 60 DEGs were upregulated in the TS vs. TP comparison and downregulated in the PS vs. PT comparison (Figure 3D, Table S5). In the cluster analysis of 2135 unigenes based on their FPKM values, PS and TS did not group together, while PS and PT did group together as did TS and TP (Figure 3E). Thus, the expression patterns of the DEGs between incompatible and compatible treatments did not group together according to pollination treatment, but rather, they grouped according to morph. These results suggest that the majority of the DEGs involved in pin compatible and self-incompatible responses differ from those involved in thrum compatible and self-incompatible responses.

### 2.4. Functional Classification of All DEGs in PS vs. PT and TS vs. TP

The DEGs from the PS vs. PT and TS vs. TP comparisons were subjected to GO enrichment analyses (Tables S7 and S8). Among the 1311 DEGs from the PS vs. PT comparison, 279, 287, and 318 were classified into the cellular component (CC), molecular function (MF), and biological process (BP) categories, respectively, while 195, 185, and 200 of the 1048 DEGs from the TS vs. TP comparison were classified into these three categories, respectively. Within the BP category, cellular process, single-organism process, and metabolic process were the most common classifications in both comparisons. Among the DEGs from both comparisons, the DEGs associated with cells, cell parts, and organelles were the most enriched in the CC category, and binding and catalytic activity were the most enriched in the MF category (Figure S5). However, in terms of the top 20 most significant terms in the BP, CC, and MF categories, clear differences were observed between the PS vs. PT and TS vs. TP comparisons (Figure S6). Directed acyclic graphs derived from the GO enrichment analysis of the BP category showed clear differences between the PS vs. PT and TS vs. TP comparisons. The TS vs. TP DEGs were mainly enriched during carbohydrate transport (GO:0008643), including monosaccharide transport (GO:0015749), hexose transport (GO:0008645), and galactose transport (GO:0015757) (Figure S7). In contrast, the PS vs. PT DEGs were mostly enriched in response to a stimulus (GO:0050896) and carbohydrate transport, including response to organonitrogen compound (GO:0010243), chitin (GO:0010200), and endogenous stimuli (GO:0009719) (Figure S8).

**Figure 4:**
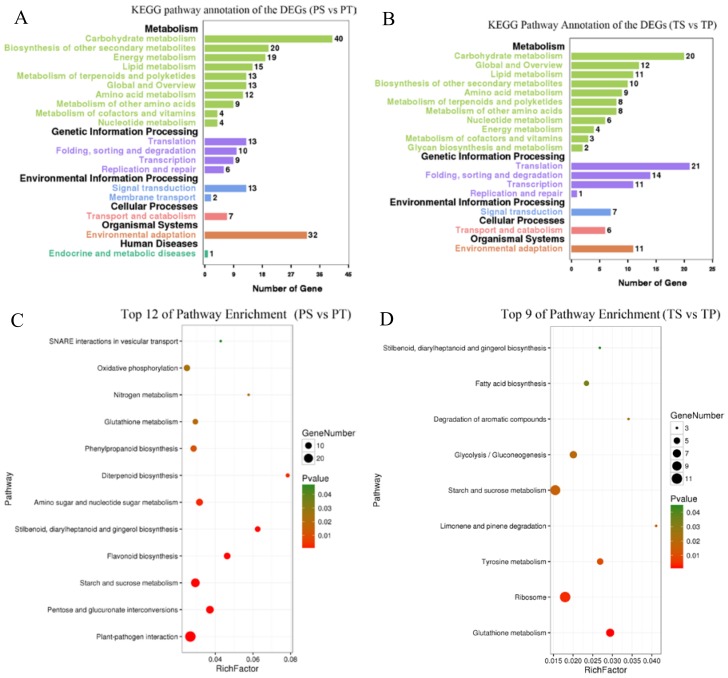
KEGG enrichment analyses of DEGs from PS vs. PT and TS vs. TP comparisons. (**A**) Number of DEGs from PS vs. PT comparison in various KEGG pathways; (**B**) Number of DEGs from TS vs. TP comparson in various KEGG pathways; (**C**) Top 12 most significantly enriched pathways in PS vs. PT comparison (*p* < 0.05); (**D**) Top nine most significantly enriched pathways in TS vs. TP comparison (*p* < 0.05). The number of genes in each category is proportional to the size of each dot, and the color of each dot represents the *p*-value.

In the KEGG enrichment analyses, 194 DEGs from the PS vs. PT comparison were categorized into 75 pathways, and 119 DEGs from the TS vs. TP comparison were categorized into 63 pathways (Tables S9 and S10). Carbohydrate metabolism, translation, and environmental adaptation were the most enriched categories containing the greatest number of DEGs, especially in the pin comparison (Figure 4A,B). Among the significantly enriched pathways (*p* < 0.05), there were more than two carbohydrate metabolism pathways in both the pin and thrum comparisons (Figure 4C,D), including starch and sucrose metabolism, pentose and glucuronate interconversions, and glycolysis/gluconeogenesis. In the TS vs. TP comparison, the most significant pathway was glutathione metabolism. In the PS vs. PT comparison, the most significant pathway was plant–pathogen interactions. There were 29 DEGs enriched in this pathway. In contrast, in the TS vs. TP comparison, nine DEGs were enriched in this pathway (Figures S9 and S10; Tables S11 and S12). Thus, the results of the KEGG and GO enrichment analyses indicated that the heteromorphic SI of *P. maximowiczii* is significantly associated with carbohydrate metabolism and stress response pathways; however, most gene functional categories differed between the PS vs. PT and TS vs. TP comparisons.

### 2.5. KEGG Enrichment Analyses of the Upregulated and Downregulated DEGs in PS vs. PT and/or TS vs. TP

**Figure 5:**
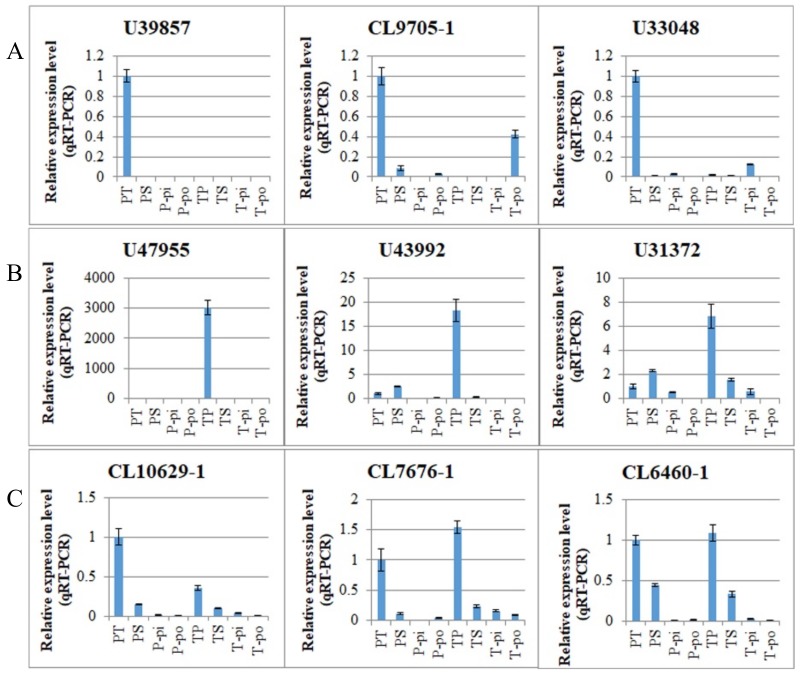
Expression patterns of DEGs downregulated in the PS vs. PT and/or TS vs. TP comparisons detected by qRT-PCR. (**A**) Expression patterns of DEGs downregulated specifically in the PS vs. PT comparison; they were expressed highly in the PT pollination; (**B**) Expression patterns of DEGs downregulated specifically in the TS vs. TP comparison; were expressed highly in the TP pollination; (**C**) Expression patterns of DEGs downregulated in the PS vs. PT and TS vs. TP comparisons; they were expressed highly in the PT and TP pollination. PT, pistils of pin after cross-pollination. PS, pistils of pin after self-pollination. P-pi, pin pistils without pollination. P-po, pin pollen. TP, pistils of thrum after cross-pollination. TS, pistils of thrum after self-pollination. T-pi, thrum pistils without pollination. T-po, thrum pollen.

In addition to the 89 DEGs with inverse expression patterns in the pin and thrum comparisons, there were 2046 DEGs (95.83%), which included 588 DEGs downregulated specifically in the PS vs. PT comparison, 432 DEGs downregulated specifically in the TS vs. TP comparison, and 115 DEGs downregulated in the PS vs. PT and TS vs. TP comparisons (Figure 3C). These downregulated DEGs exhibited increased expression levels in compatible pollinations (PT and TP) compared to those in incompatible pollinations (PS and TS) in the same morphs (Tables S3, S4 and S6). In order to investigate the functional classifications of these DEGs, the three groups of downregulated DEGs were subjected to the KEGG enrichment analysis. As described above (Section 2.4), the analysis of all DEGs from the PS vs. PT and TS vs. TP comparisons showed that carbohydrate metabolism, translation, and environmental adaptation were the most significantly enriched categories and that significant categories differed between the PS vs. PT and TS vs. TP comparisons. The KEGG enrichment analysis of the three groups of downregulated DEGs returned similar results. The pathways associated with carbohydrate metabolism and environmental adaptation were the most significantly enriched among downregulated DEGs specific to the PS vs. PT comparison, while translation was the most enriched category among the downregulated DEGs specific to the TS vs. TP comparison (Figure S11A,B). Furthermore, DEGs downregulated in both the PS vs. PT and TS vs. TP comparisons were significantly enriched during environmental adaptation (Figure S11C). The most significant pathway among downregulated DEGs specific to the PS vs. PT comparison was plant–pathogen interactions (Table 2), and the most significant pathway among the downregulated DEGs specific to the TS vs. TP comparison was glutathione metabolism (Table 3). Similarly, significantly enriched pathways (*p* < 0.05) among the DEGs downregulated in the PS vs. PT and TS vs. TP comparisons included plant–pathogen interactions and glutathione metabolism (Table 4). Three DEGs from each of these three groups of downregulated DEGs were selected for qRT-PCR analysis in various tissues, including the pistils after cross- and self-pollination, the pistils without pollination, and the pollens of pin and thrum morphs. The results were consistent with the RNA-seq data, and the Pearson’s r in the linear fitting analysis of the expression levels in qRT-PCR and RNA-seq was 0.9458 (Figures S12 and S16). There was higher expression in the compatible pollinations than in the incompatible pollinations and no specific expression in the pistils or pollen (Figure 5).

**Figure 6:**
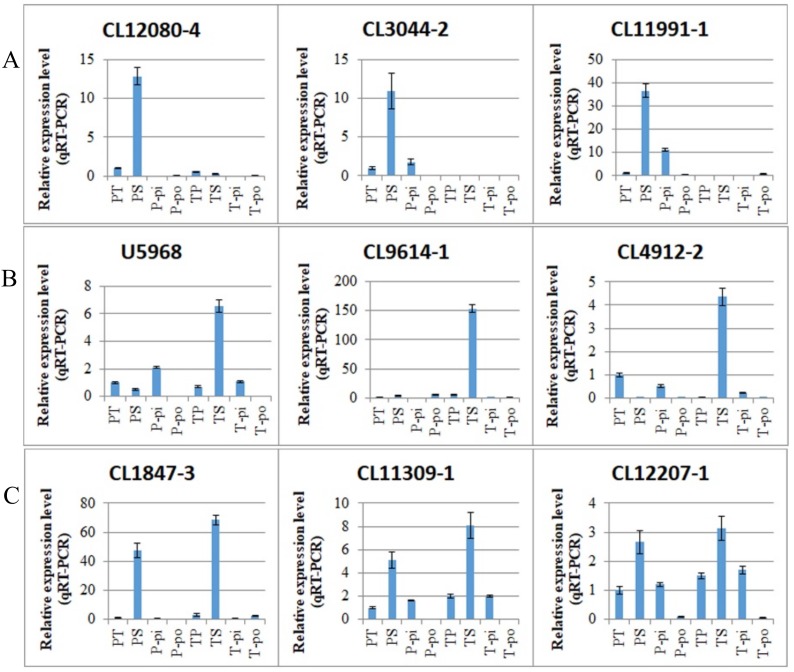
Expression patterns of DEGs upregulated in the PS vs. PT and/or TS vs. TP comparisons detected by qRT-PCR. (**A**) Expression patterns of DEGs upregulated specifically in the PS vs. PT comparison which were expressed highly in PS pollination; (**B**) Expression patterns of DEGs upregulated specifically in the TS vs. TP comparison which were expressed highly in TS pollination; (**C**) Expression patterns of DEGs upregulated in the PS vs. PT and TS vs. TP comparisons which were expressed highly in PS and TS pollination. PT, pistils of pin after cross-pollination. PS, pistils of pin after self-pollination. P-pi, pin pistils without pollination. P-po, pin pollen. TP, pistils of thrum after cross-pollination. TS, pistils of thrum after self-pollination. T-pi, thrum pistils without pollination. T-po, thrum pollen.

In contrast to the downregulated DEGs, among the 2046 DEGs, there were 499 that were specifically upregulated in the PS vs. PT comparison, 392 that were specifically upregulated in the TS vs. TP comparison, and 20 that were upregulated in both the PS vs. PT and TS vs. TP comparisons (Figure 3C). The upregulated DEGs showed higher expression levels in the incompatible pollinations (PS and TS) than in the compatible pollinations (PT and TP) in the same morphs (Tables S3, S4 and S6). The KEGG enrichment analysis indicated that the upregulated and downregulated DEGs exhibited some similarities and some dissimilarities in terms of functional classification. Like the downregulated DEGs, the upregulated DEGs from the PS vs. PT and TS vs. TP comparisons were mostly enriched during carbohydrate metabolism and translation, respectively (Figure S13A,B), and many DEGs were associated with environmental adaptation. In addition, many DEGs upregulated specifically in the PS vs. PT comparison were associated with energy mechanisms. The most significant pathway among upregulated DEGs specific to the PS vs. PT comparison was the pentose and glucuronate interconversions pathway (Table 5), and the most significant pathway among upregulated DEGs specific to the TS vs. TP comparison was tyrosine metabolism (Table 6). We selected three DEGs from each of the three groups of upregulated DEGs for qRT-PCR analysis in various tissues. The qRT-PCR results confirmed the expression patterns derived from the RNA-seq analysis (Figure S14). They also confirmed the upregulation of these DEGs in incompatible pollinations vs. compatible pollinations and showed no specific expression in the pistils or pollen (Figure 6).

Thus, it appears that the genes that may be helpful for promoting the compatibility or incompatibility of the involvement of *P. maximowiczii* in carbohydrate metabolism, environmental adaptation, and translation. However, the DEGs from the PS vs. PT comparison differed from those in the TS vs. TP comparison, and the DEGs that were downregulated and upregulated in the compatible pollinations partially differed from those in the incompatible pollinations.

### 2.6. Genes That Are Differentially Expressed in a Genotype-Specific Manner in Pollen

**Figure 7:**
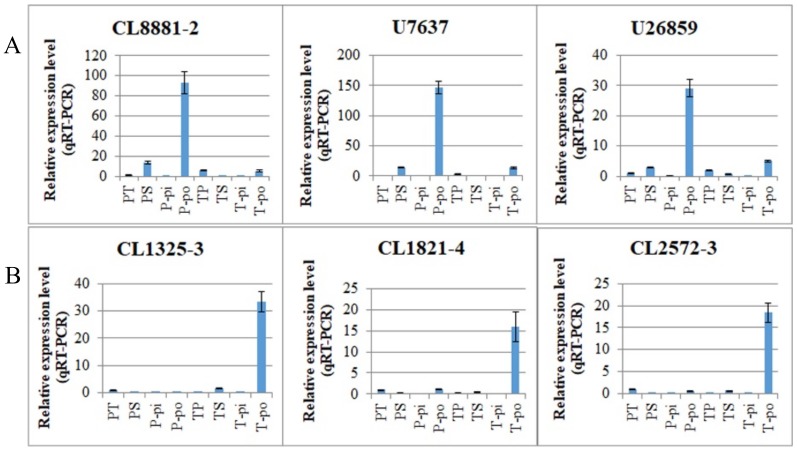
Expression patterns of DEGs with inverse expression patterns in PS vs. PT and TS vs. TP comparisons detected by qRT-PCR. (**A**) DEGs upregulated in the PS vs. PT comparison and downregulated in the TS vs. TP comparison were highly expressed in pin pollen; (**B**) DEGs downregulated in the PS vs. PT comparison and upregulated in the TS vs. TP comparison were highly expressed in thrum pollen. PT, pistils of pin after cross-pollination. PS, pistils of pin after self-pollination. P-pi, pin pistils without pollination. P-po, pin pollen. TP, pistils of thrum after cross-pollination. TS, pistils of thrum after self-pollination. T-pi, thrum pistils without pollination. T-po, thrum pollen.

In this study, the DEGs associated with pin and thrum SI were identified by comparing PS with PT and TS with TP. This experimental design could, however, cause some DEGs to be identified based on their differential mRNA expressions in the mature pollen of the pin and thrum morphs, as the pollen genotype differed between the two combinations, even though the pistil genotype was constant. As described above, we found that 89 (4.17%) of the 2135 DEGs showed inverse expression patterns in the two comparisons. Among these, 29 DEGs were upregulated in the PS vs. PT comparison and downregulated in the TS vs. TP comparison, and 60 DEGs were downregulated in the PS vs. PT comparison and upregulated in the TS vs. TP comparison (Table S6). This may be associated with differences in the pollen genotype, as the same expression levels were observed in the treatments involving the same type of pollen; for example, there were 29 DEGs with high expression in both PS and TP which were both pollinated with pin pollen, and 60 DEGs with high expression in both PT and TS which were both pollinated with thrum pollen. Thus, we speculate that there are 29 DEGs associated with the pin pollen genotype and 60 DEGs associated with the thrum pollen genotype. The 29 pin pollen DEGs included polyol transporter 5 (*PLT5*), pectinesterase 50 (*PME50*), stress-induced protein KIN2 (*KIN2*), cytochrome P450 704C1 (*CYP704C1*), polygalacturonase (*PG1*), and pathogenesis-related protein PR-1-like (*PR1*). The 60 thrum pollen DEGs included putative RALF-like gene (*RALF-like*), beta-d-xylosidase 5 (*BXL5*), beta-fructofuranosidase, insoluble isoenzyme CWINV1 (*CWINV1*), sugar transport protein, auxin-induced protein, and GDSL esterase (Table S6). We selected three DEGs from each of these two groups for qRT-PCR analysis in various tissues. The results confirmed the expression patterns derived from the RNA-seq analysis (Figure S15). Furthermore, they showed significantly elevated expression levels in pollen compared to other tissues. *U7637*, *CL881-2*, and *U26859*, which were upregulated in the PS vs. PT comparison and downregulated in the TS vs. TP comparison, were highly expressed in pin pollen, while *CL1821-4*, *CL1325-3*, and *CL2572-3*, which were downregulated in the PS vs. PT comparison and upregulated in the TS vs. TP comparison, were highly expressed in thrum pollen (Figure 7).

### 2.7. Transcriptomics Profiles of DEGs Associated with Plant-Pathogen Interactions

**Figure 8:**
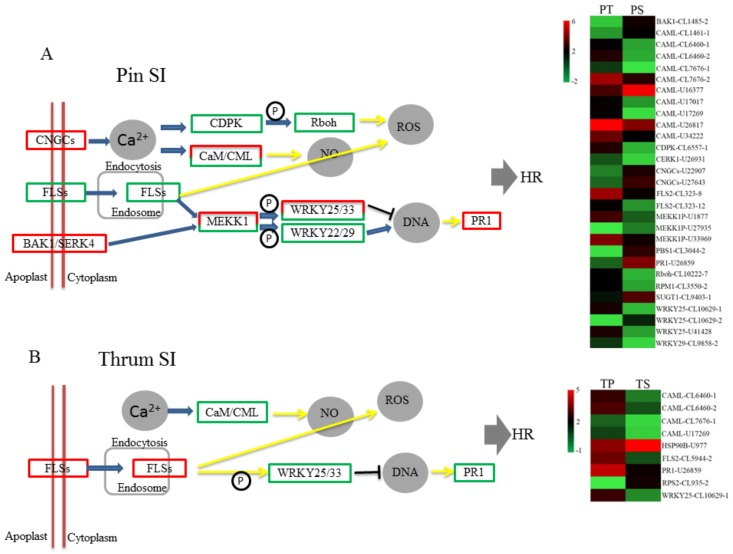
Analysis of DEGs enriched in plant–pathogen interaction pathways. (**A**) Simplified diagram of plant–pathogen interaction pathway of DEGs from the PS vs. PT comparison and the expression levels of the DEGs in the PT and PS pollinations. (**B**) Simplified diagram of the plant–pathogen interaction pathway of DEGs from the TS vs. TP comparison and the expression levels of the DEGs in the TP and TS pollinations. The green frame outlines indicate nodes with downregulated DEGs; red outlines indicate nodes with upregulated DEGs; green and red outlines indicate nodes with both up and downregulated DEGs. The colour scale represents the log2 values (the values of the expression levels of the genes).

Based on the KEGG analysis, 29 DEGs from the PS vs. PT comparison and nine DEGs from the TS vs. TP comparison were enriched in plant–pathogen interaction pathways (Figure S9 and S10; Table S11 and S12). The simplified pathway graphs of plant-pathogen interactions were derived from the original KEGG enrichment analysis maps (Figure 8A,B). The graphs showed that the plant–pathogen interaction pathway appeared to be more complex in the pin SI process than in the thrum SI process. PS vs. PT DEGs were enriched in the CDPK, Rboh, Cam/CML, MEKK1, WRKY, PR1, and transmembrane KO nodes, including CNGCs, FLS2, and BAK1 (Figure 8A), while TS vs. TP DEGs were only enriched in the Cam/CML, WRKY, PR1, and FLS2 nodes (Figure 8B). Among these, there were 10 CAML DEGs from the PS vs. PT comparison but only four from the TS vs. TP comparison, and most of the putative CAML genes were downregulated in the SI responses. There were five DEGs from PS vs. PT enriched in transmembrane KO nodes, including two putative *CNGC*, two putative *FLS2* and one putative *BAK1*. However, only one DEG from TS vs. TP, one different putative *FLS2*, was present in the transmembrane FLS2 nodes (Figure 8A,B). Thus, it appears that the transcriptomics profiles of the DEGs enriched in plant-pathogen interaction pathways are quite divergent between the PS vs. PT and TS vs. TP comparisons.

### 2.8. Transcriptomics Analysis of Transcription Factors (TFs) in P. maximowiczii SI

TFs are believed to play important roles in controlling many secondary metabolic processes. We identified 33 TFs from the 2135 DEGs using the PlantTFDB database. Among them, 12 TFs were differentially expressed in PS and PT, 11 were differentially expressed in TS and TP, and 10 were differentially expressed in the compatible and self-incompatible responses of both the pin and thrum morphs (Table S13 and S14). Most TFs belonged to the ERF family (11), followed by the C2H2 (7), WRKY (4), MYB-related (2), and NAC (2) families. The ERF, C2H2, and WRKY families are known to be involved in plant defense responses, abiotic stress, and other stress responses [32,33,34,35,36], but have rarely been associated with SI in previous studies. Our study demonstrates that these genes are differentially expressed in the heteromorphic SI of *P. maximowiczii* and that most of them are downregulated in self-pollinations (Figure S18).

## 3. Discussion

Compared to GSI and SSI, the molecular mechanisms of heteromorphic SI are poorly understood. The SI mating types of *Primula* are thought to be controlled by a diallelic S locus [8]. Some genes in the S locus of *Primula* have been reported recently [10,18]; however, the female and male determinants of heteromorphic SI, the signaling components activated after the interaction of determinants, and the events involved in the process of heteromorphic SI remain unknown.

SSI systems usually take the form of stigmatic inhibition of self-pollen, and GSI systems frequently show pollen inhibition in the style [37,38]. Therefore, although heteromorphic SI has been traditionally classified as sporophytic, it has been hypothesized that the SI mechanism of the thrum morph is sporophytic while that of the pin morph may be both sporophytic and gametophytic [2]. In our study, self-pollen tubes of the thrum morph rarely reached the style, while the self-pollen tubes of the pin morph occasionally, though rarely, entered the style. Therefore, our results support this hypothesis. Furthermore, based on our transcriptomics analyses, about 90% of the 2135 DEGs identified between the self- and cross-pollinated pistils were specifically expressed in either the pin (PS vs. PT) or thrum (TS vs. TP) morphs. Moreover, the GO and KEGG enrichment analyses demonstrated that the genes involved in the pin SI process were distinct from those involved in thrum SI. Based on these results, we hypothesize that the pin and thrum SI responses differ and have different underlying molecular mechanisms.

Carbohydrate metabolism participates in many plant biological processes. Our transcriptomics data showed that the DEGs identified in the heteromorphic SI process of *P. maximowiczii* were significantly enriched in those involved in carbohydrate metabolism. A total of 17 downregulated DEGs and 24 upregulated DEGs were associated with carbohydrate metabolism (Figures S11 and S13). Furthermore, the results of the GO and KEGG enrichment analyses for all DEGs highlighted the responses to stimuli and environmental adaptation categories as important in the processes of compatibility and incompatibility, and the analyses of upregulated and downregulated DEGs returned similar results. Notably, the most significantly enriched KEGG pathway in the PS vs. PT comparison was plant-pathogen interactions which were enriched with 29 DEGs from the PS vs. PT comparison and nine DEGs from the TS vs. TP comparison. Based on our analysis, TFs, ERF, C2H2, and WRKY proteins were identified as instrumental in the SI process and are known to be involved in plant defense and other stress responses. In conclusion, the bioinformatics analysis of the transcriptomics data suggested that the pathways associated with environmental adaptation are involved in heteromorphic SI, especially those involved in pathogen defense. Thus, the heteromorphic SI response of *P. maximowiczii* may be associated with both environmental adaptation and carbohydrate metabolism.

In the plant-pathogen interaction pathway, we observed that three KO nodes enriched for DEGs act across the cell membrane, namely, CNGCs, BAK1/SERK4, and FLSs (Figure 8). It is known that the female S determinants, SRK in SSI and S-RNase in GSI, in several plant species (Brassicaceae, Solanaceae, and Papaveraceae) are transmembrane proteins containing transmembrane domains [39,40,41,42,43]. The genes encoding these transmembrane proteins may participate in the recognition of the female and male S determinants of *P. maximowiczii* SI. In the FLSs node, there were enriched with the DEGs of both PS vs. PT and TS vs. TP, but the DEGs were different and showed different expression patterns. In the PS vs. PT comparison, two FLS DEGs, both putative polygalacturonase inhibitors (PGIPs), were implicated, while in the TS vs. TP comparison, one FLS DEG, a putative endoglucanase (EG), was implicated. It has been reported that PGIPs play important roles in the defense against pathogenic fungi and pollen development [44,45] and that EGs are associated with the degradation of the cell wall and pollen tube growth [46,47]. Thus, the putative PGIP and EG proteins may participate in the processes of pin and thrum SI, respectively. In the future, we plan to investigate the function of these DEGs in more detail and to identify the key genes involved in the recognition processes of pin and thrum SI.

In our study, we found that 89 (4.17%) of the 2135 DEGs showed inverse expression patterns in the two comparisons and may therefore be associated with the pollen genotype. Therefore, this raises the question of whether these DEGs are involved in SI or whether they are simply associated with pollen morphology. For example, one unigene, predicted to be a putative RALF-like gene (*CL2572-3*), was found to exhibit inverse expression patterns between PS vs. PT and TS vs. TP comparisons. It was downregulated in PS vs. PT, with a fold change >9 and upregulated in TS vs. TP with a fold change of seven (Table S13). We have confirmed this gene is specifically expressed in thrum pollen (Figure 7B). The RALF-like gene has been previously reported in *P. vulgaris* as a pollen-expressed gene and was expressed earlier in development in thrum pollen than in pin pollen [48]. Moreover, RALF-like genes can alter the pH of the cell and play a role in regulating plant development [49]; cytosolic pH acidification has been confirmed as an integral and essential event for the SI response in *Papaver rhoeas* [50]. This raises the question of whether this putative RALF-like gene is involved in the *Primula* SI response. One putative PME gene (*CL8881-2*) was differentially expressed in the PS vs. PT and TS vs. TP comparisons and is specifically expressed in pin pollen (Figure 7A). PMEs are involved in the degradation of the cell wall, pollen tube growth, and plant defense mechanisms against pathogens [51,52,53,54]. In addition, there are known male S determinant genes of SI systems that are specifically expressed in the anther or pollen, such as *SP11/SCR* in *Brassica* and *SLF* in *Antirrhinum* and *Prunus* [40,55,56,57]. However, determining whether these genes that are associated with pollen genotype participate in the *Primula* SI response or interact with the male S determinant gene requires additional research.

After removing the 89 DEGs with inverse expression patterns in the pin and thrum comparisons, there were 2046 DEGs (95.83%), including the upregulated and downregulated DEGs specific to the TS vs. TP and PS vs. PT comparisons and the upregulated and downregulated DEGs that were present in both the TS vs. TP and PS vs. PT comparisons. Nine hundred and eleven were upregulated and 1135 were downregulated in the self-pollinations vs. the cross-pollinations (Figure 3C). The genes with different expression patterns were specific to the different pollination combinations. For example, among the genes tested, the upregulated DEGs specific to the PS vs. PT comparison were expressed specifically in the PS treatment. They showed a higher expression level in the PS pollination than in the other pollinations and no specific expression in the pistils or pollen. This result suggests that the genes are associated with the incompatible response of pin morph. Similarly, the upregulated DEGs specific to the TS vs. TP comparison were expressed specifically in the TS pollination, and the downregulated DEGs specific to the PS vs. PT comparison were expressed specifically in the PT pollination and so on. In the cross-pollinated pistils, the pollen germinated and grew into the ovary, while in the self-pollinated plants, most pollen grains or pollen tubes were inhibited on the stigma surface or in the stigma. As the downregulated DEGs were more highly expressed in compatible combinations than in incompatible combinations, downregulated DEGs may be associated with the compatibility response. As described above, downregulated DEGs involved in carbohydrate metabolism may be involved in supplying the energy for pollen tube growth. In contrast, upregulated DEGs were more highly expressed in the incompatible combinations. Some of these DEGs were also involved in carbohydrate metabolism and energy metabolism (Figure S13). However, even if the downregulated DEGs play a role in supplying energy to compatible pollen tubes, the functions of the upregulated DEGs associated with carbohydrate metabolism and energy metabolism in the SI response remain unknown. Moreover, how do these genes regulate the compatible or incompatible responses? Does this mean that silencing of the highly expressed genes would inhibit the compatible or incompatible responses? Alternatively, if the lowly expressed genes were overexpressed, would the compatible or incompatible response be promoted? Our study provides interesting data that may help to answer these questions and is a basis for further investigations into the molecular mechanisms of *Primula* SI.

## 4. Materials and Methods

### 4.1. Plant Material

After *P. maximowiczii* entered dormancy in October, plants were stored at −2 °C for more than 60 days and then cultivated in a peat and perlite mixture (3:1) in 10 cm × 10 cm plastic pots in phytotron [58]. The air temperature was 20 °C, and the photoperiod was long-day condition with a 16-h light and 8-h dark cycle. The peat was 10–30 mm sphagnum peat (Pindstrup, Ryomgaard, Denmark), with a pH of 6.0 and the value of Electrical Conductivity (EC) of 0.8 mS cm^−1^. The plants were watered once per week and fertilized with 2 g L^−1^ general purpose water-soluble fertilizer (EVERRIS, Dublin, OH, USA) once every two weeks.

### 4.2. Artificial Pollination

To investigate fruiting rates, four pollination treatments between pin and thrum morphs were conducted, including pin cross-pollination (PT), pin self-pollination (PS), thrum cross-pollination (TP), and thrum self-pollination (TS). Ten healthy plants at similar growth stages were selected as maternal parents, and at least 50 flowers per treatment were pollinated. Three biological repeats were obtained for each combination. Flowers were emasculated one day before anthesis by the removal of the corolla and anthers, and then they were bagged in paper bags. Anthers were collected at the same time and dried at 25 °C for 24 h. Pollen was collected and stored at −20 °C in bottles containing desiccants. Cross-pollinations were performed using pollens from different morphs, while self-pollination was performed using pollen from the same plant. The fruiting rate per pollination treatment was calculated as the percentage of pollinated flowers that developed into fruits with seeds.

### 4.3. Pollen Germination and Pollen-Tube Growth In Vivo

Four pollination treatments were conducted as above. Five pistils from each treatment group were collected at 2, 4, 6, 8, 12, 24, 48, 72, 96, 144, 168, and 192 h after pollination and were treated with fixative (three parts ethanol and one part glacial acetic acid) for at least 24 h, softened in 8 mol L^−1^ NaOH at 25 °C for 10 min, washed briefly three times in water, and stained with 0.1% water-soluble aniline blue dissolved in 0.1 mol K_3_PO_4_ for 1 h [59]. The pistils were assessed for pollen germination and pollen tube growth using a LEICA M165 FC fluorescent microscope (LEICA, Buffalo Grove, IL, USA) with a LEICA DFC450 C digital camera (LEICA, Buffalo Grove, IL, USA).

### 4.4. RNA Isolation, RNA-seq, and De Novo Transcriptome Assembly

Based on the results above, four pollination combinations were compared: PT, PS, TP, and TS. Fifteen pistils were collected at 8, 24, and 48 h after pollination, and the 45 pistils of each combination were mixed together as one sample to ensure the presence of incompatible and compatible responses in the pistils. Three biological replicates were obtained for each combination. All samples for RNA isolation were flash-frozen in liquid nitrogen and stored at −80 °C. Total RNA was extracted and purified using the CTAB reagent. Magnetic beads with Oligo (dT) were used to enrich and isolate mRNA. Then, cDNA libraries were constructed, and an Agilent 2100 Bioanalyzer was used for qualification of the sample library. The libraries were sequenced using an Illumina HiSeq™ 2000, and de novo assembly was performed with the Trinity program. Transcripts <200 bp were removed, and the longest transcript from each locus was selected as the unigene. Please see Methods S1 for the analytic methods used for the raw data. The raw RNA-seq data were deposited in the NCBI’s Sequence Read Archive (SRA) under the accession number SRP135553.

### 4.5. Gene Annotation and DEG Analysis

Six databases were used to annotate unigenes and predict their coding regions, including the GO, COG, KEGG, Swiss-Prot, nr, and nt databases (Methods S1). RPKM (reads per kilobase of transcript per million mapped reads) was used to calculate and normalize the expression levels of unigenes [60]. DEGs between different treatments were identified using the following criteria: probability ≥ 0.8 and |(log_2_ fold change)| ≥ 1. DEGs were used for KEGG and GO enrichment analyses [61]. Candidate transcription factors were predicted with PlantTFDB (available online: http://planttfdb.cbi.pku.edu.cn/, accessed on 8 December 2017) [62].

### 4.6. Real-Time PCR Assays

Various plant tissue samples were used for the extraction of total RNA using an RNAprep Pure Plant Kit (TIANGEN, Beijing, China), and RNA was used to synthesize single-stranded cDNA with the PrimeScript RT Reagent Kit (Takara, Shiga, Japan). The samples included pin pistils after cross-pollination (PT), pin pistils after self-pollination (PS), pin pistils without pollination (P-pi), pin pollen (P-po), thrum pistils after cross-pollination (TP), thrum pistils after self-pollination (TS), and thrum pistils without pollination (T-pi), thrum pollen (T-po). Real-time PCR was performed with SYBR Green I dye (Takara, Japan). The relative expression levels of genes were calculated using the 2^−ΔΔCt^ method with the *actin* gene as an internal control. The *tubulin* gene was used as the negative control gene. The primers used for qPCR are provided in the supporting information (Table S15). qPCR was performed with the following parameters: initial denaturation at 95 °C for 30 s; 40 cycles of 95 °C for 5 s and 60 °C for 30 s; followed by a melting-curve stage of 95 °C for 15 s, 60 °C for 1 min, and 95 °C for 15 s. Each reaction consisted of 4.7 μL sterile distilled water, 0.4 μL each of 10 μM forward and reverse primers, 2 μL of first-strand cDNA, and 7.5 μL of SYBR Premix *Ex Taq*. To validate the correlation of the qRT-PCR results and the RNA-seq data, the scatter diagrams and the linear fitting analysis were performed using OriginPro 9.1 software (OriginLab, Northampton, MA, USA).

## 5. Conclusions

Using transcriptomics analyses, the current study investigated the fruit setting rates of compatible and self-incompatible pollinations of *P. maximowiczii* as well as the growth and inhibition of pollen tubes and the expression patterns of genes involved in pin and thrum SI using RNA-seq. Our results indicate that the SI systems of the pin and thrum morphs may be controlled by different molecular mechanisms and that the heteromorphic SI process of *P. maximowiczii* may be associated with carbohydrate metabolism and environmental adaptation. These results provide new insight into the SI process of *Primula* and promote further research into the molecular mechanisms of heteromorphic SI.

## Figures and Tables

**Table 1:** Numbers of unigenes functionally annotated with different databases.

Database	Number of Unigenes	Percentage (%)
Non-Redundant protein sequence (NR)	59,798	59.95
Nucleotide Sequence Database (NT)	48,174	48.29
Swiss-Prot	39,419	39.52
Kyoto Encyclopedia of Genes and Genomes (KEGG)	36,120	36.21
Cluster of Orthologous Groups of proteins (COG)	25,123	25.81
Gene Ontology (GO)	45,456	45.57
With annotations	61,552	61.70
Without annotations	38,202	38.30
Total unigenes	99,754	100.00

**Table 2:** KEGG terms enriched among downregulated DEGs specific to the PS vs. PT comparison.

KEGG Class	Pathway	*p*-Value
Environmental adaptation	Plant–pathogen interaction	0.0002
Carbohydrate metabolism	Amino sugar and nucleotide sugar metabolism	0.0008
Biosynthesis of other secondary metabolites	Stilbenoid, diarylheptanoid and gingerol biosynthesis	0.0014
Biosynthesis of other secondary metabolites	Flavonoid biosynthesis	0.0025
Biosynthesis of other secondary metabolites	Phenylpropanoid biosynthesis	0.0120
Amino acid metabolism	Cysteine and methionine metabolism	0.0190
Metabolism of other amino acids	Glutathione metabolism	0.0199
Metabolism of terpenoids and polyketides	Diterpenoid biosynthesis	0.0206
Folding, sorting, and degradation	The soluble *N*-ethylmaleimide-sensitive factor attachment protein receptor (SNARE) interactions in vesicular transport	0.0371
Metabolism of terpenoids and polyketides	Carotenoid biosynthesis	0.0399
Lipid metabolism	Cutin, suberine, and wax biosynthesis	0.0493

**Table 3:** KEGG terms enriched among downregulated DEGs specific to the TS vs. TP comparison.

KEGG Class	Pathway	*p*-Value
Metabolism of other amino acids	Glutathione metabolism	0.0026

**Table 4:** KEGG terms enriched among downregulated DEGs in the PS vs. PT and TS vs. TP comparisons.

KEGG Class	Pathway	*p*-Value
Lipid metabolism	Fatty acid biosynthesis	0.0009
Global and overview	Fatty acid metabolism	0.0024
Environmental adaptation	Plant-pathogen interaction	0.0060
Metabolism of other amino acids	Glutathione metabolism	0.0291

**Table 5:** KEGG terms enriched among upregulated DEGs specific to the PS vs. PT comparison.

KEGG Class	Pathway	*p*-Value
Carbohydrate metabolism	Pentose and glucuronate interconversions	0.0001
Energy metabolism	Oxidative phosphorylation	0.0005
Biosynthesis of other secondary metabolites	Flavonoid biosynthesis	0.0050
Metabolism of terpenoids, and polyketides	Diterpenoid biosynthesis	0.0280
Energy metabolism	Nitrogen metabolism	0.0290
Energy metabolism	Carbon fixation in photosynthetic organisms	0.0369
Translation	Ribosome	0.0369
Replication and repair	Nucleotide excision repair	0.0458
Carbohydrate metabolism	Starch and sucrose metabolism	0.0471

**Table 6:** KEGG terms enriched among upregulated DEGs specific to the TS vs. TP comparison.

KEGG Class	Pathway	*p*-Value
Amino acid metabolism	Tyrosine metabolism	0.0119
Biosynthesis of other secondary metabolites	Isoquinoline alkaloid biosynthesis	0.0157
Translation	Ribosome	0.0193
Carbohydrate metabolism	Citrate cycle (tricarboxylic acid (TCA) cycle)	0.0327
Folding, sorting, and degradation	Proteasome	0.0371

## Supplementary Materials

Supplementary materials can be found at http://www.mdpi.com/1422-0067/19/7/1840/s1.
