# Supplementary material for: Transcriptomics Investigation into the Mechanisms of Self-Incompatibility between Pin and Thrum Morphs of Primula maximowiczii
Source: Int J Mol Sci. 2018 Jun 22;19(7):1840. doi: 10.3390/ijms19071840 (PMC6073747; doi:10.3390/ijms19071840)
Supplement: Supplementary file 1 [file ijms-19-01840-s001.zip › Supplementary files-2018.06.12/Figure S1.pdf]

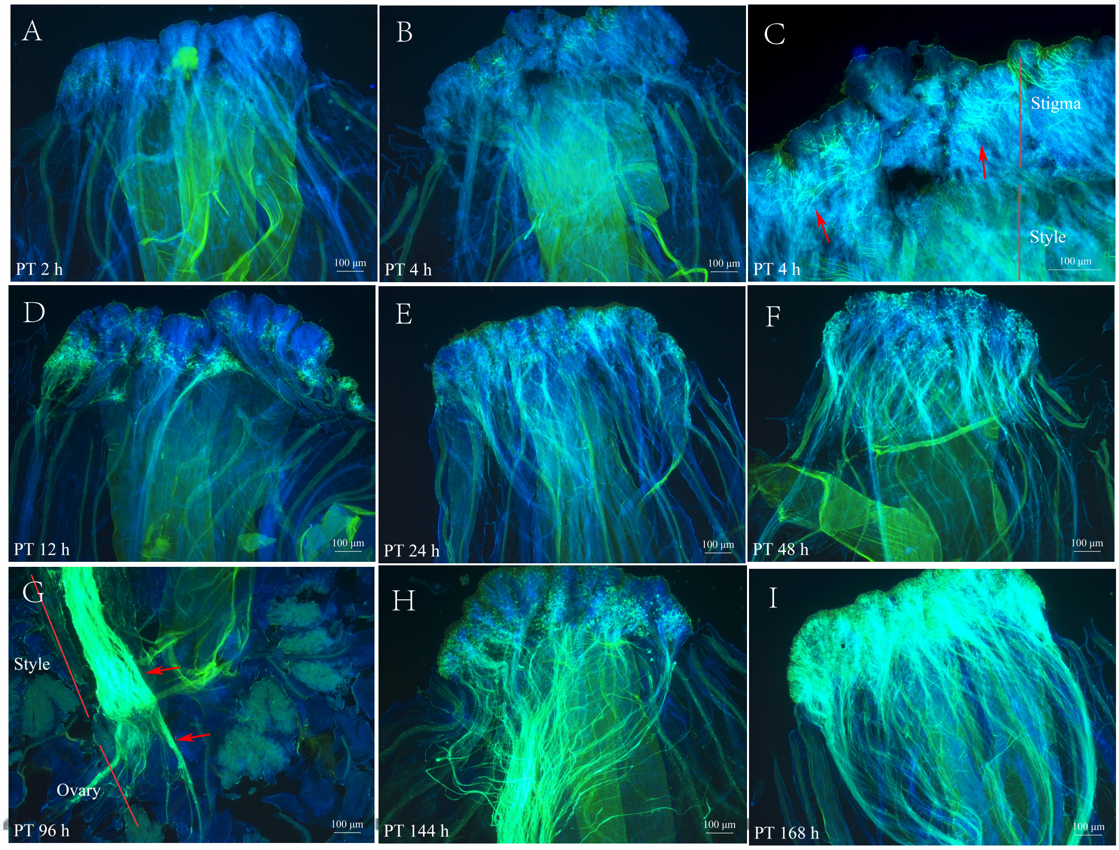

Figure S1. Pollen germination and pollen tube growth of PT at 2 h, 4 h, 12 h, 24 h, 48 h, 96 h, 144 h and 168 h after pollination. In PT, pollen grains started to germinate at 4 h and the pollen tubes grew in bundle in the styles at 8 h, 12 h, 24 h and so on. Then the pollen tubes entered the ovary at 96 h after pollination.
