# Supplementary material for: Transcriptomics Investigation into the Mechanisms of Self-Incompatibility between Pin and Thrum Morphs of Primula maximowiczii
Source: Int J Mol Sci. 2018 Jun 22;19(7):1840. doi: 10.3390/ijms19071840 (PMC6073747; doi:10.3390/ijms19071840)
Supplement: Supplementary file 1 [file ijms-19-01840-s001.zip › Supplementary files-2018.06.12/Figure S3.pdf]

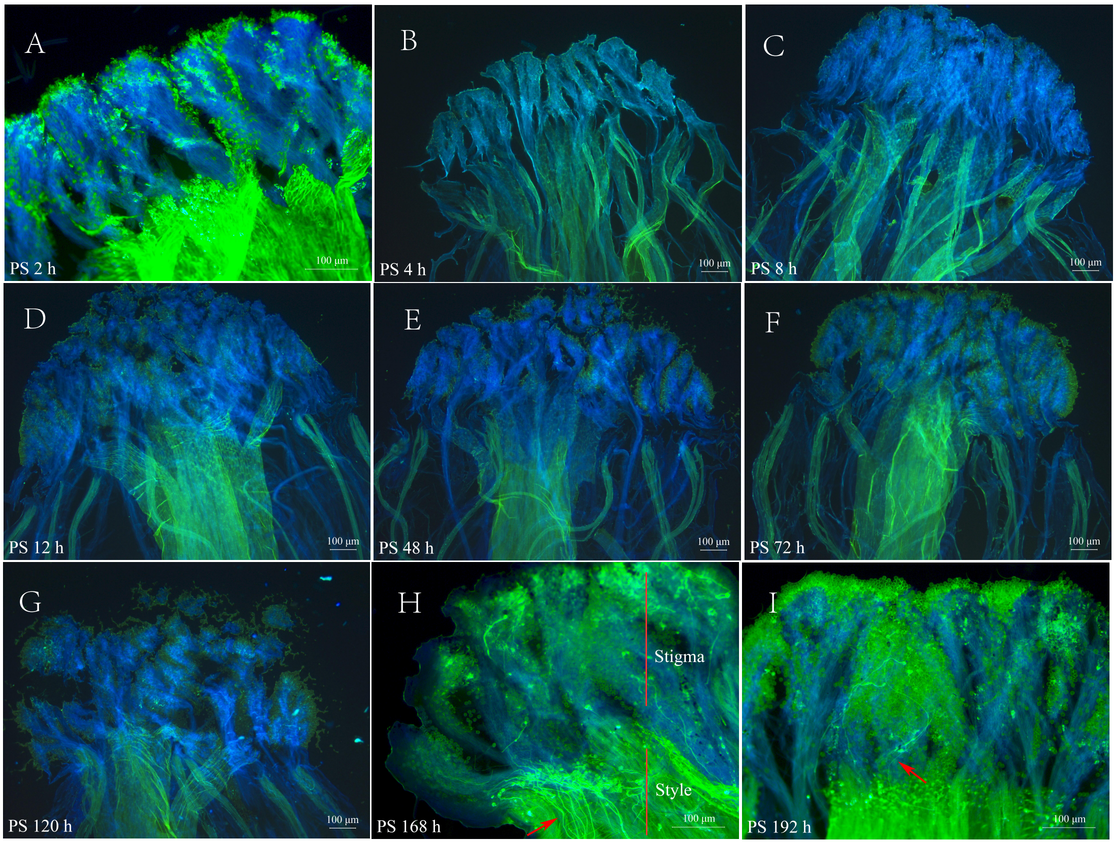

Figure S3. Pollen germination and pollen tube growth of PS at 2 h, 4 h, 8 h, 12 h, 48 h, 72 h, 120 h, 168 h and 192 h after pollination. In PS, the germinations of pollen grains were almost not observed except at 168 h and 192 h. Although only a few pollen tubes, they grew across the stigmas and entered the pin styles at 168 h and 192 h.
