# Supplementary material for: Transcriptomics Investigation into the Mechanisms of Self-Incompatibility between Pin and Thrum Morphs of Primula maximowiczii
Source: Int J Mol Sci. 2018 Jun 22;19(7):1840. doi: 10.3390/ijms19071840 (PMC6073747; doi:10.3390/ijms19071840)
Supplement: Supplementary file 1 [file ijms-19-01840-s001.zip › Supplementary files-2018.06.12/Figure S4.pdf]

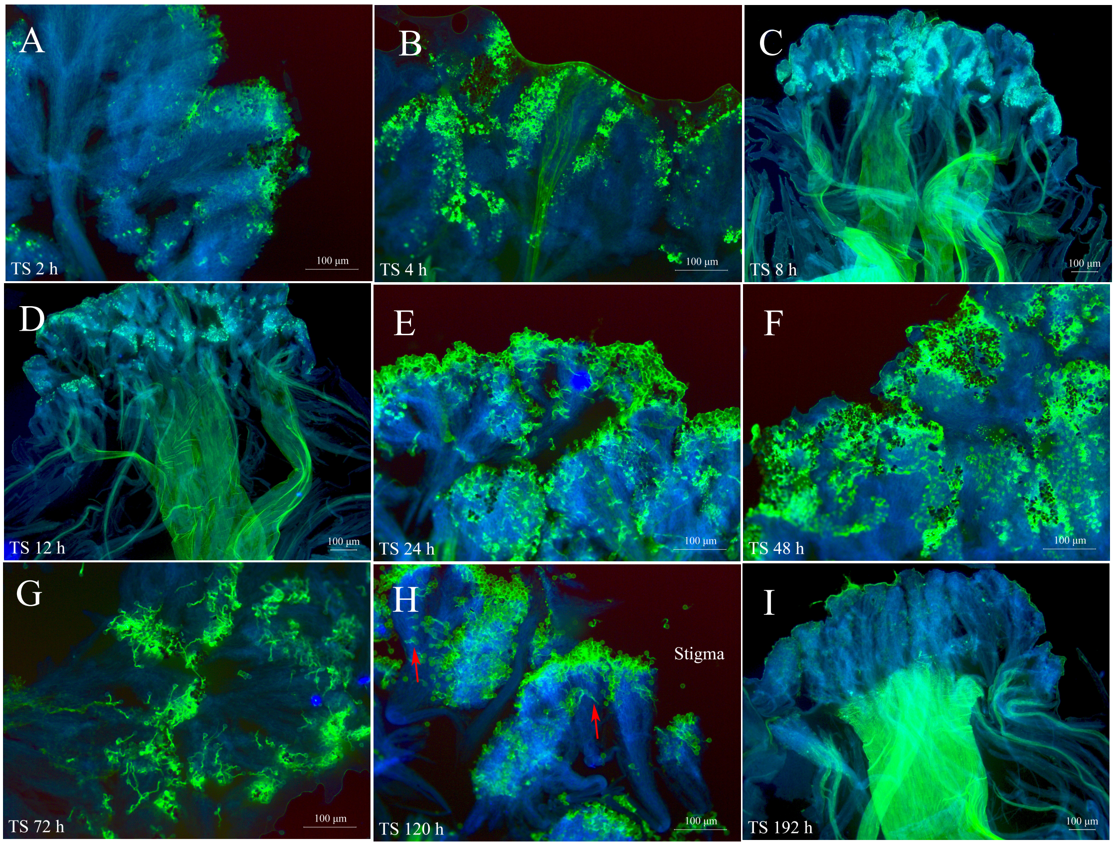

Figure S4. Pollen germination and pollen tube growth of TS at 2 h, 4 h, 8 h, 12 h, 24 h, 48 h, 72 h, 120 h and 192 h after pollination. In TS, the germination of pollen grains on the stigmas were observed at most of times. The pollen tubes could grow into stigmas but they were not observed to enter the styles.
