# Supplementary material for: Transcriptomics Investigation into the Mechanisms of Self-Incompatibility between Pin and Thrum Morphs of Primula maximowiczii
Source: Int J Mol Sci. 2018 Jun 22;19(7):1840. doi: 10.3390/ijms19071840 (PMC6073747; doi:10.3390/ijms19071840)
Supplement: Supplementary file 1 [file ijms-19-01840-s001.zip › Supplementary files-2018.06.12/Figure S5-S18.pdf]

A

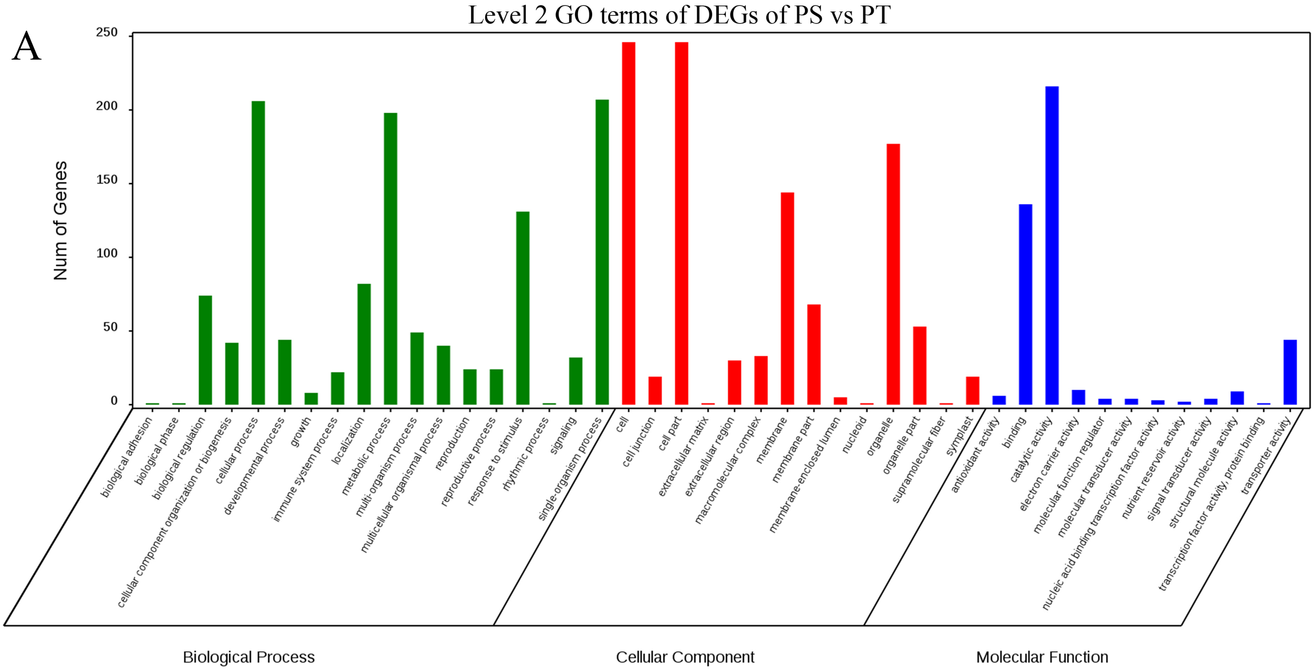

B

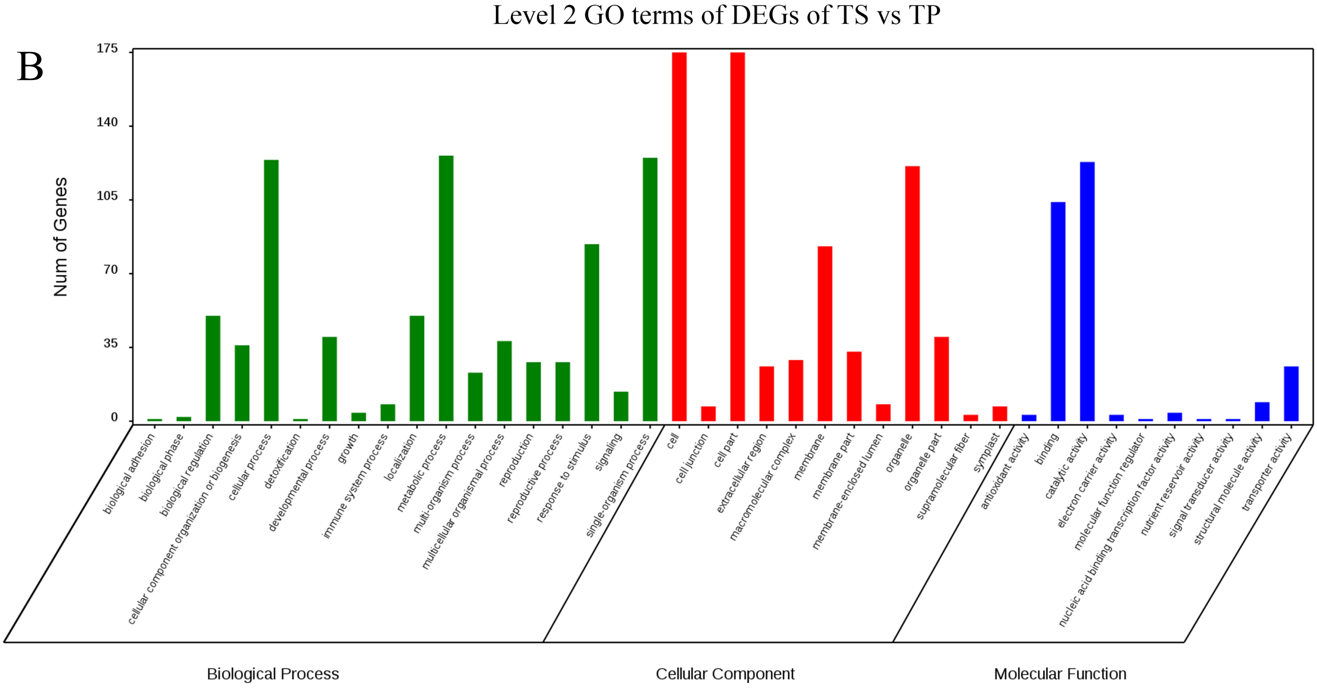

Figure S5. Gene Ontology classification of the DEGs. (A) GO classification of the DEGs from the PS vs PT comparison. (B) GO classification of the DEGs from the TS vs TP comparison.

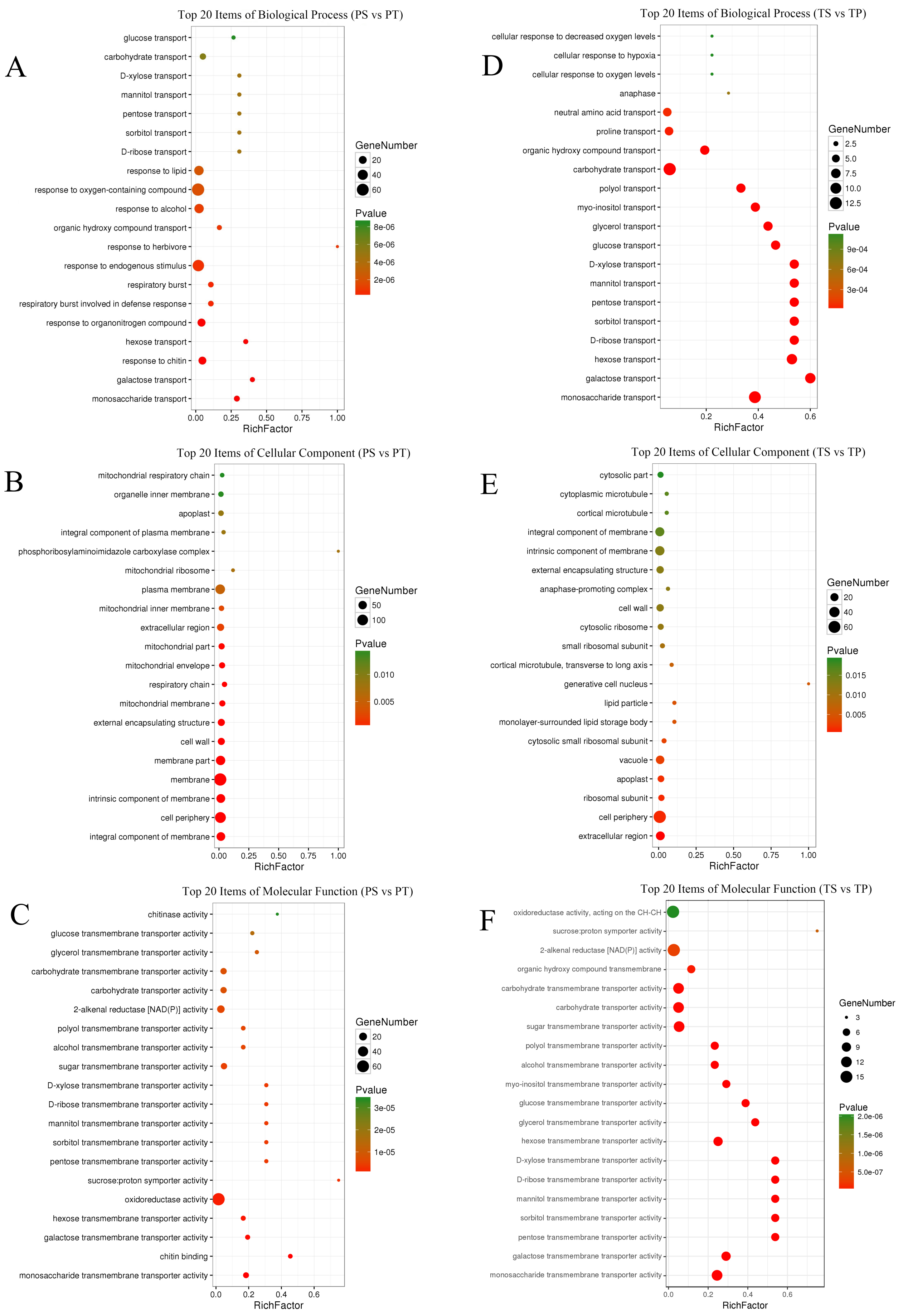

Figure S6. GO enrichment analysis of the DEGs. (A) ,(B) and (C) Top 20 items of three categaries enriched with the DEGs from the PS vs PT comparison. (D) ,(E) and (F) Top 20 items of three categaries enriched with the DEGs from the TS vs TP comparison. The enrichment results of pin and thrum DEGs were very different.

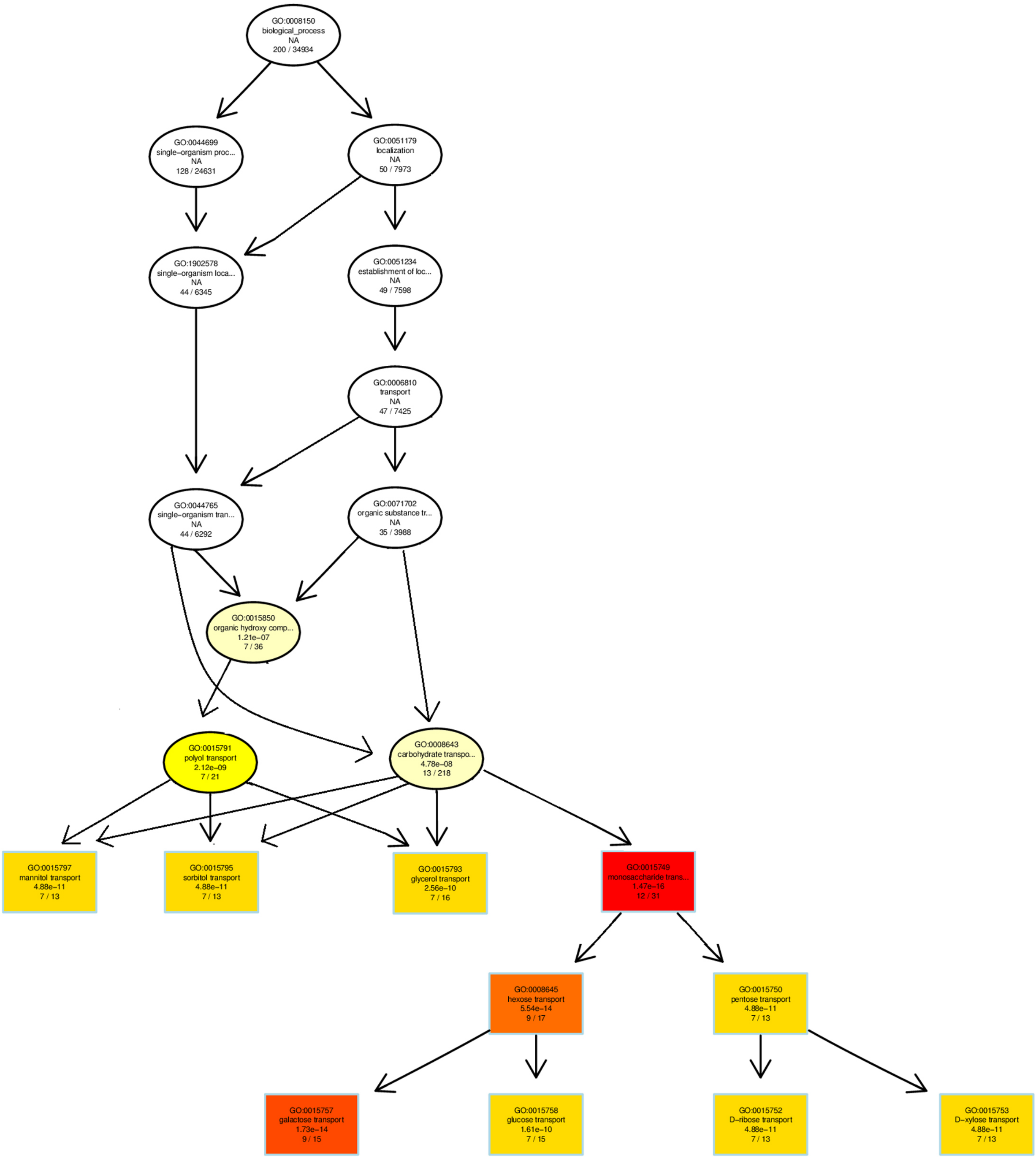

Figure S7. The directed acyclic graph of biological process of the DEGs from the TS vs TP comparison.

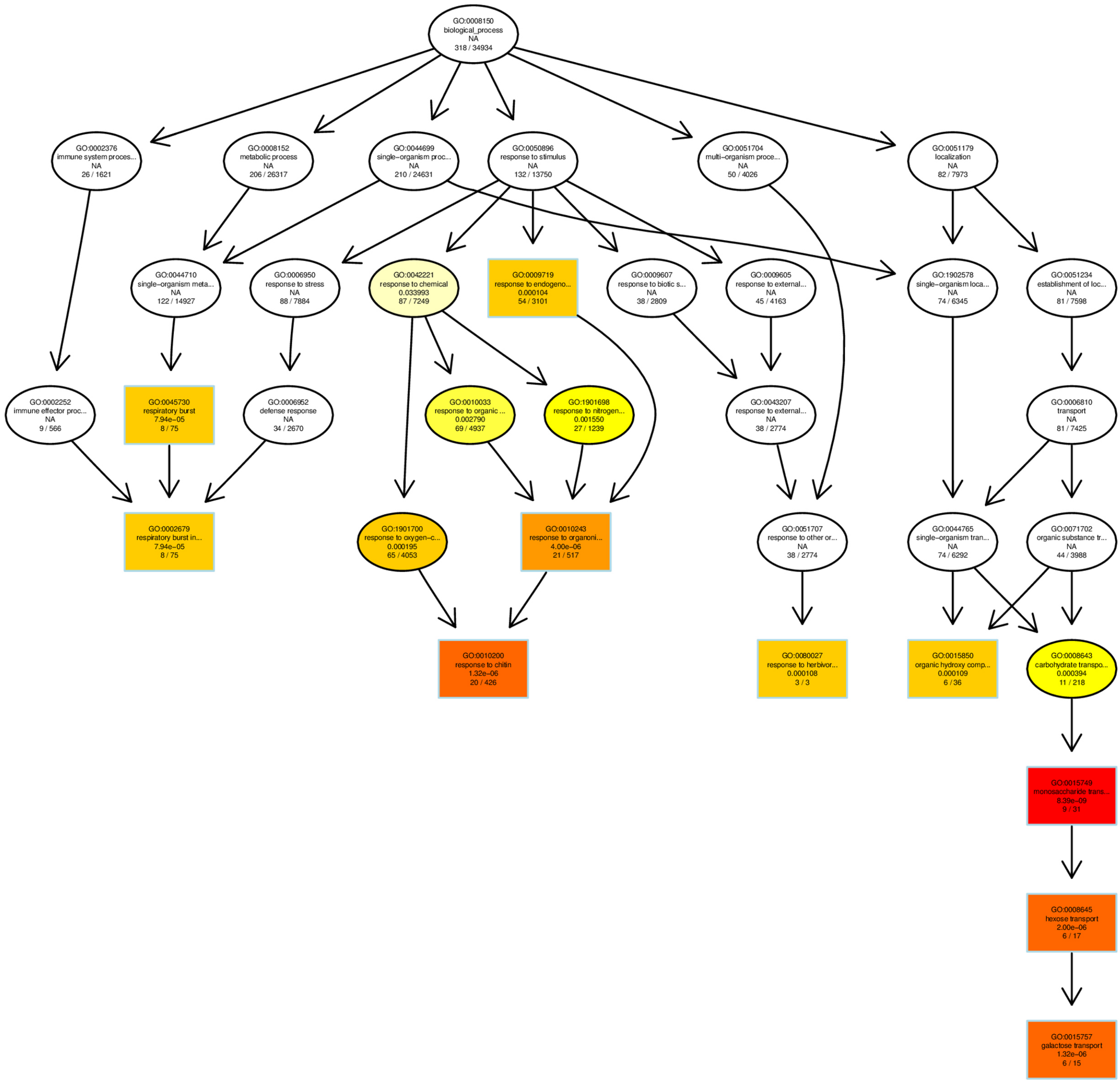

Figure S8. The directed acyclic graph of biological process of the DEGs from the PS vs PT comparison.

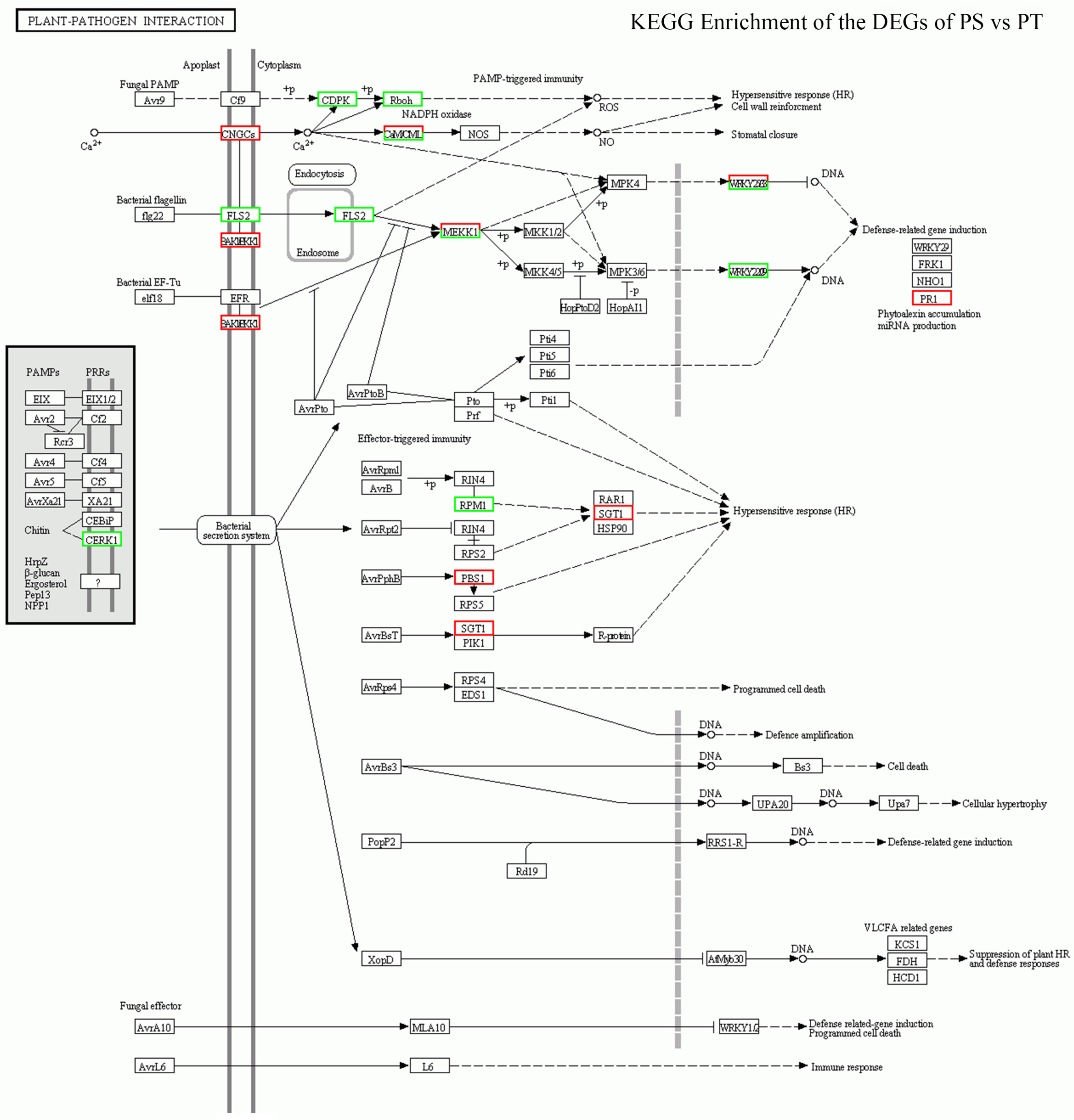

Figure S9. The maps of plant-pathogen interaction enriched with the DEGs of PS vs PT. Green frame means the en yme with the down-regulated DEGs red frame means the enzyme with up-regulated DEGs green and red frame means the enzyme with up-regulated and down-regulated DEGs.

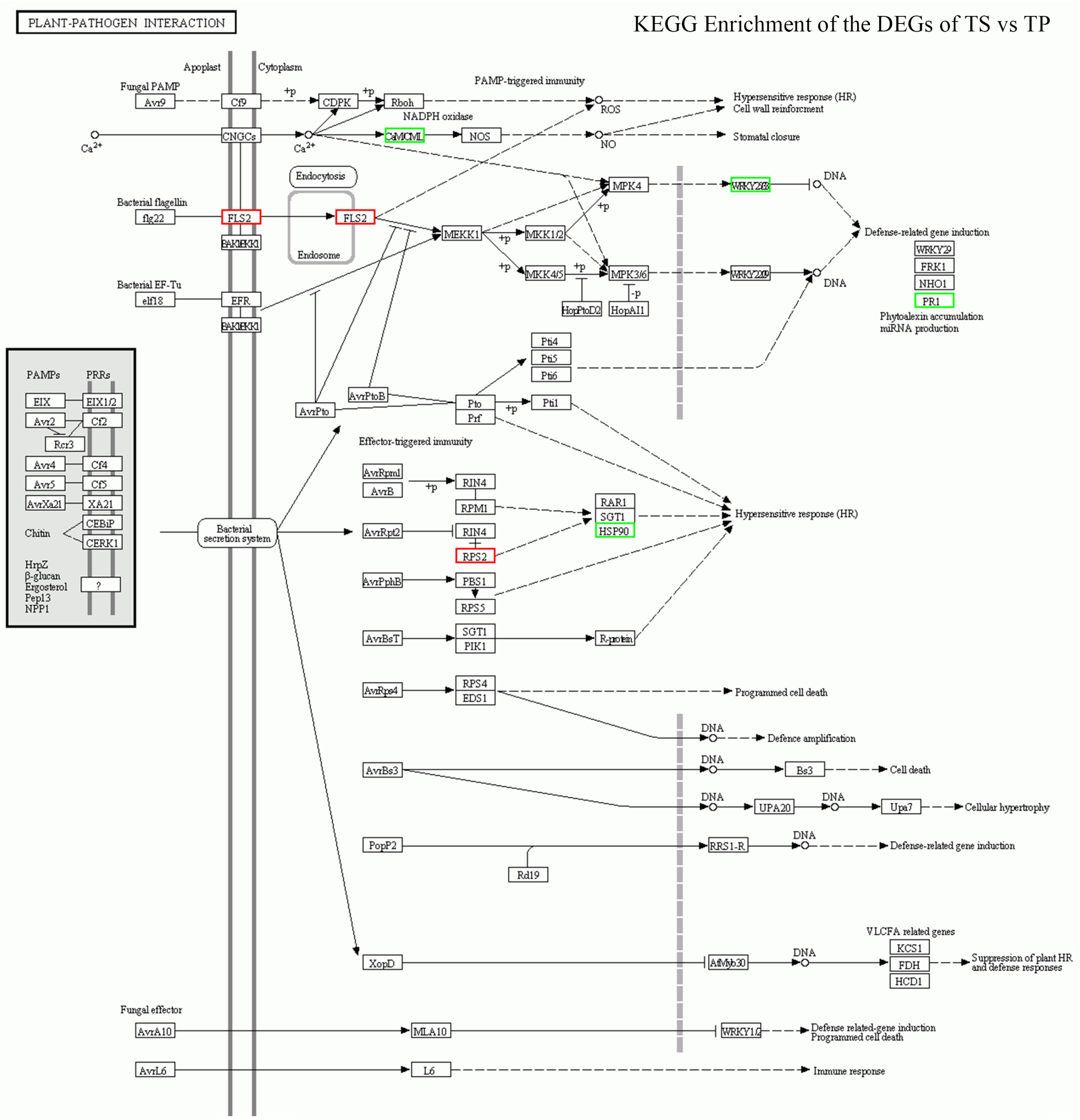

Figure S10. The maps of plant-pathogen interaction enriched with the DEGs from the TS vs TP comparison. Green frame means the enzyme with the down-regulated DEGs, red frame means the enzyme with up-regulated DEGs, green and red frame means the enzyme with up-regulated and down-regulated DEGs.

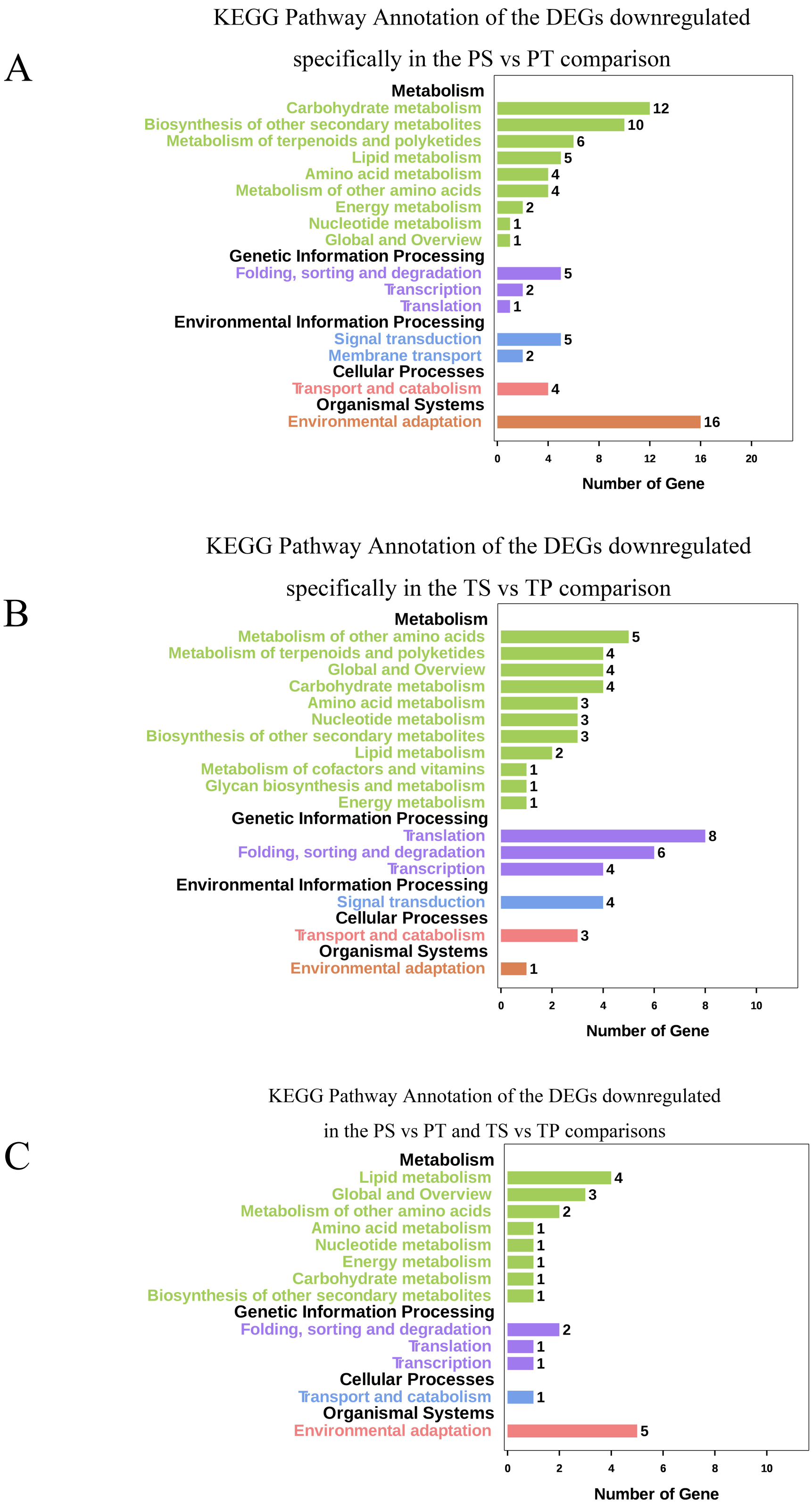

Figure S11. KEGG enrichment analyses of DEGs downregulated in the PS vs PT and/or TS vs TP comparisons. (A) The numbers of genes enriched in KEGG pathways from the DEGs downregulated specifically in the PS vs PT comparison. (B) The numbers of genes enriched in KEGG pathways from the DEGs downregulated specifically in the TS vs TP comparison. (C) The numbers of genes enriched in KEGG pathways from the DEGs downregulated in the PS vs PT and TS vs TP comparisons.

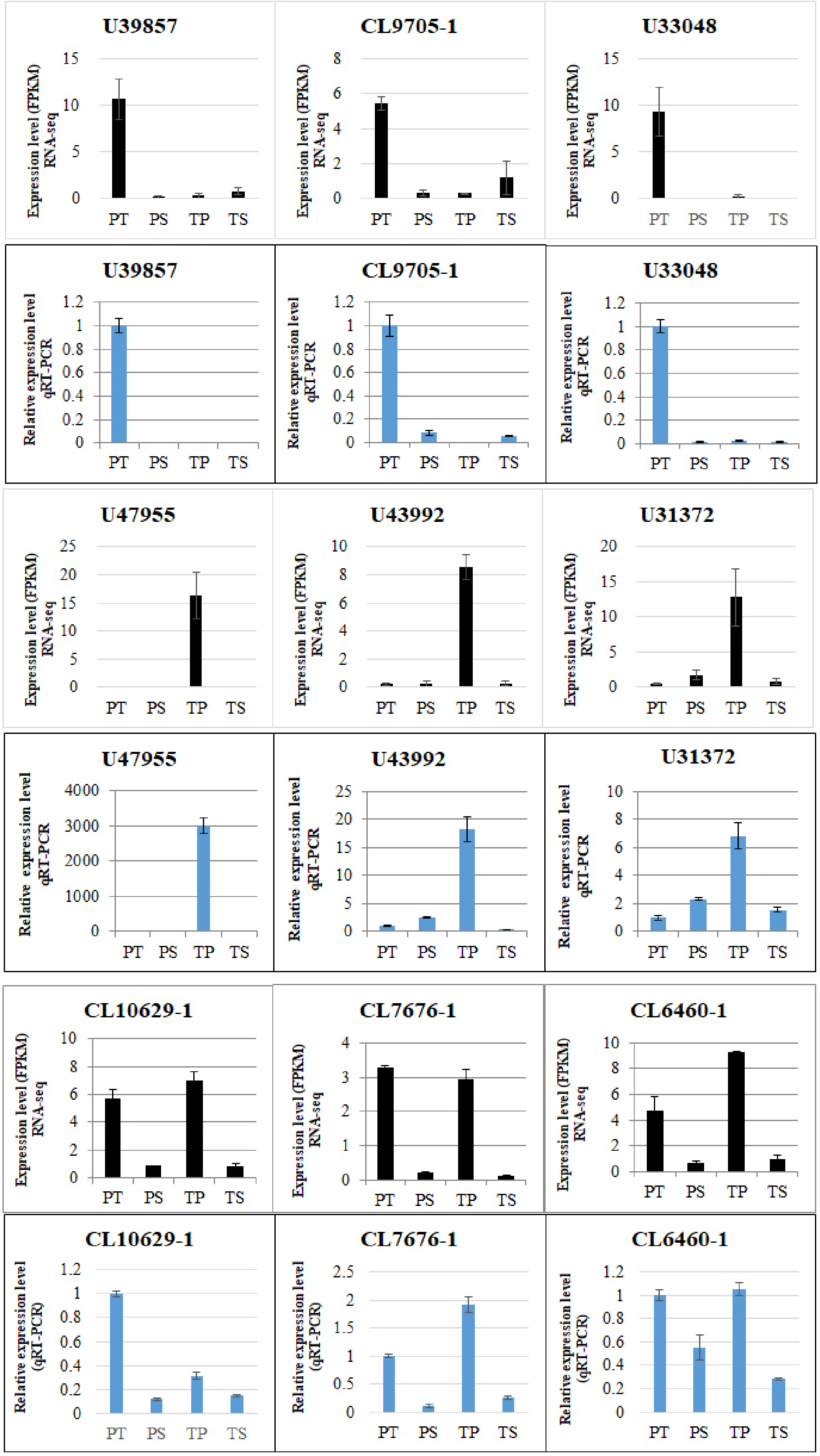

Figure S12. Expression patterns of DEGs downregulated in the PS vs PT and/or TS vs TP comparisons detected by qRT-PCR and RNA-seq. The qRT-PCR results confirmed the expression patterns derived from RNA-seq analysis.

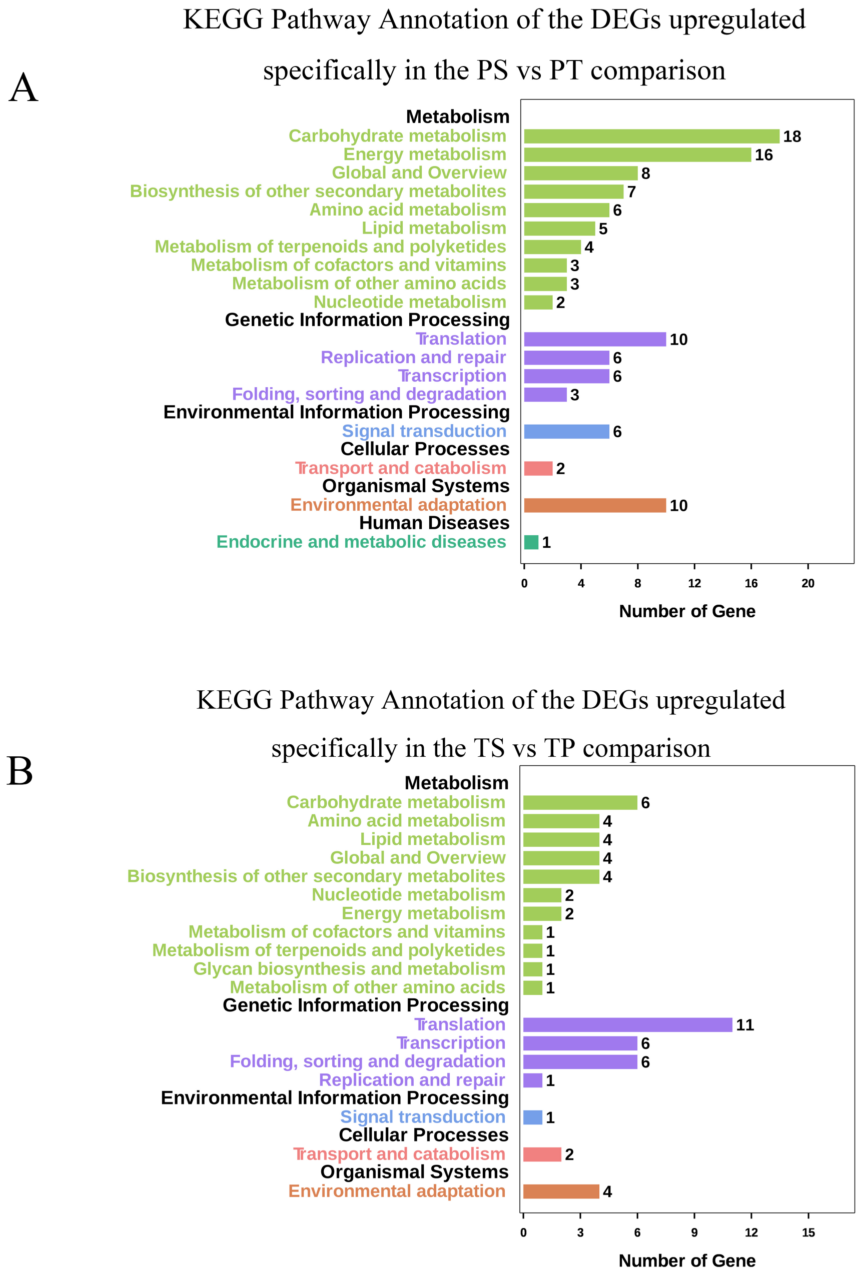

Figure S13. KEGG enrichment analyses of DEGs upregulated specifically in the PS vs PT or TS vs TP comparisons. (A) The numbers of genes enriched in KEGG pathways from the DEGs upregulated specifically in the PS vs PT comparison. (B) The numbers of genes enriched in KEGG pathways from the DEGs upregulated specifically in the TS vs TP comparison.

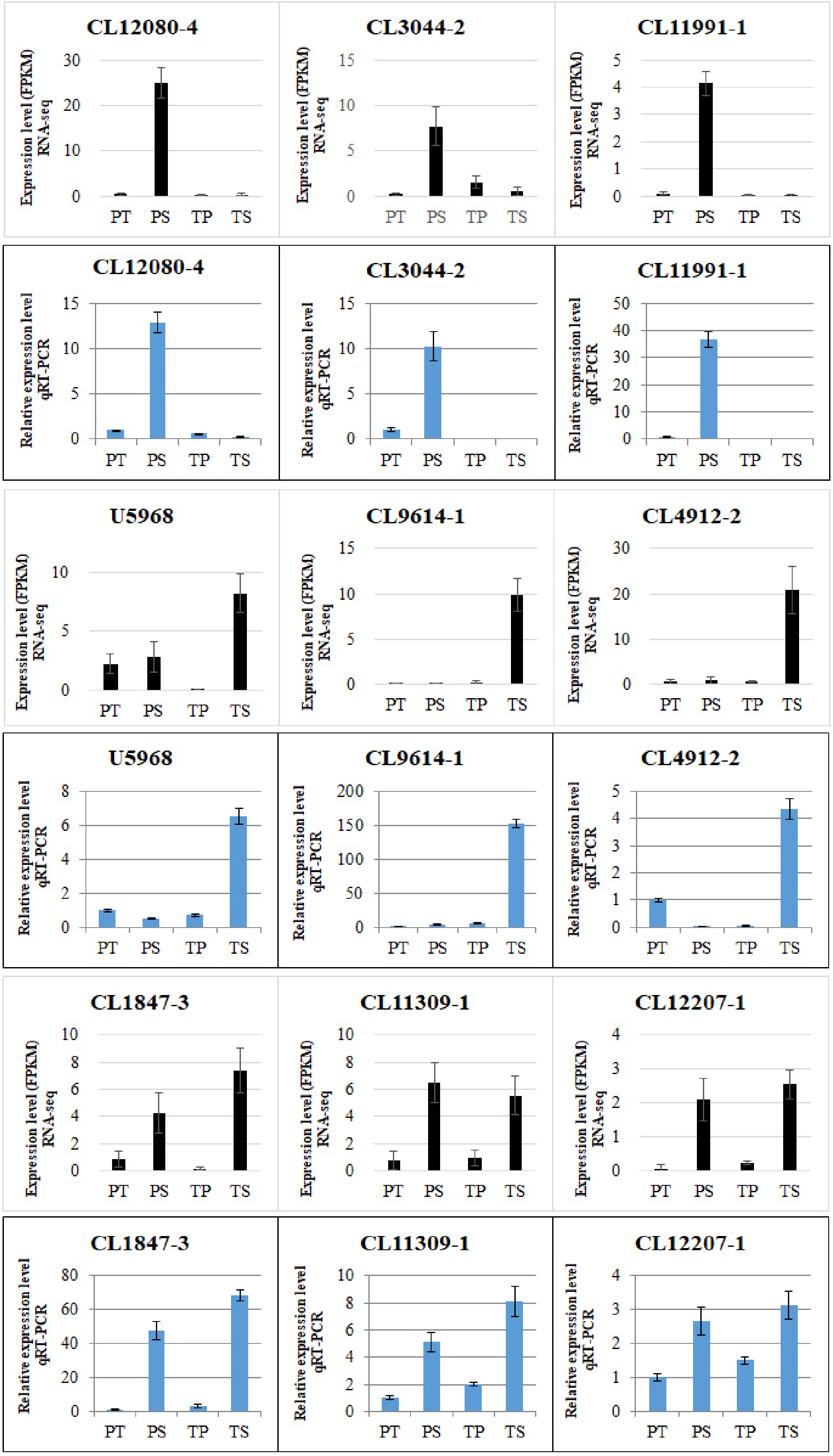

Figure S14. Expression patterns of DEGs upregulated in the PS vs PT and/or TS vs TP comparisons detected by qRT-PCR and RNA-seq. The qRT-PCR results confirmed the expression patterns derived from RNA-seq analysis.

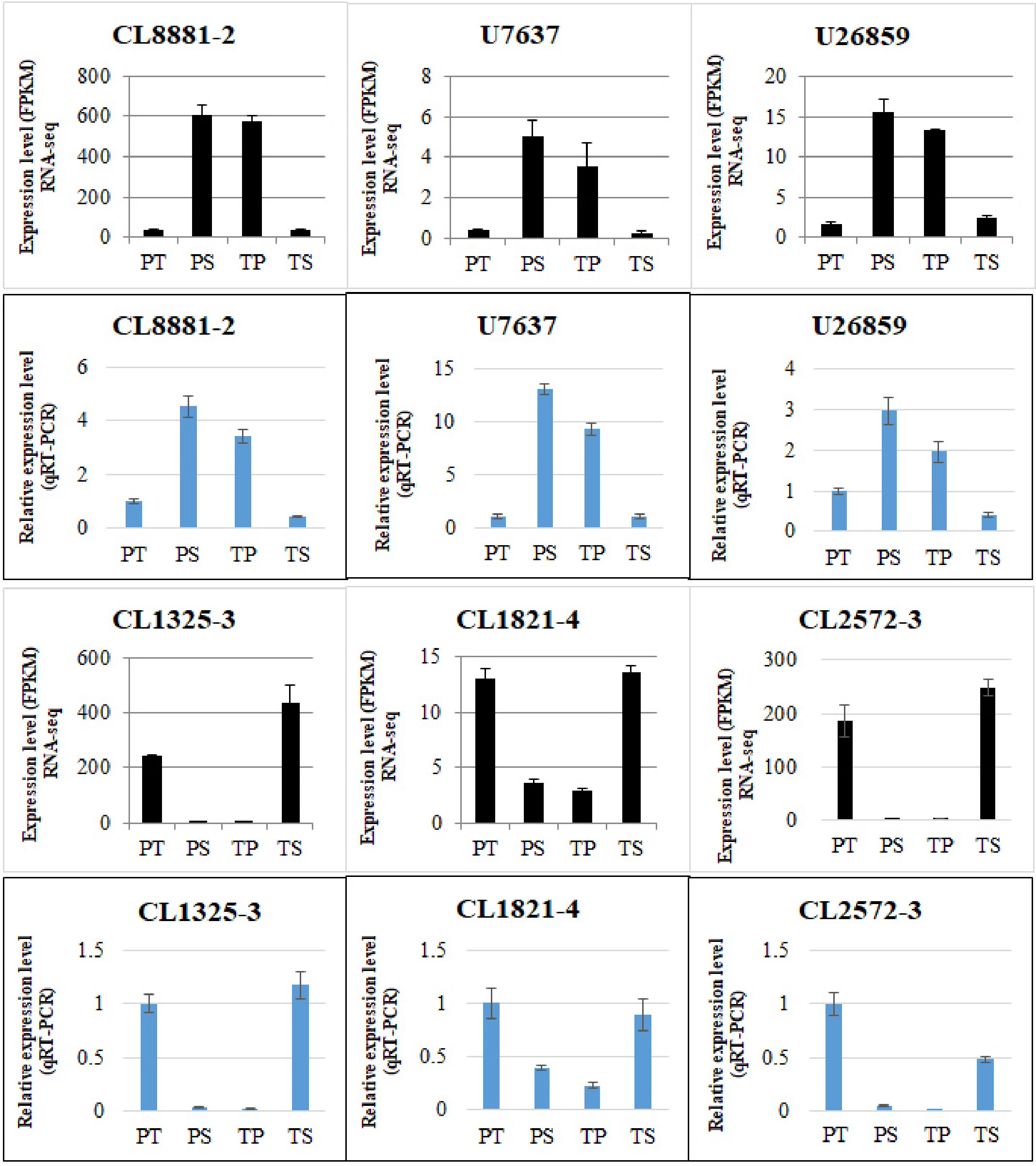

Figure S15. Expression patterns of DEGs with inverse expression patterns in the PS vs PT and TS vs TP comparisons detected by qRT-PCR and RNA-seq. The qRT-PCR results confirmed the expression patterns derived from RNA-seq analysis.

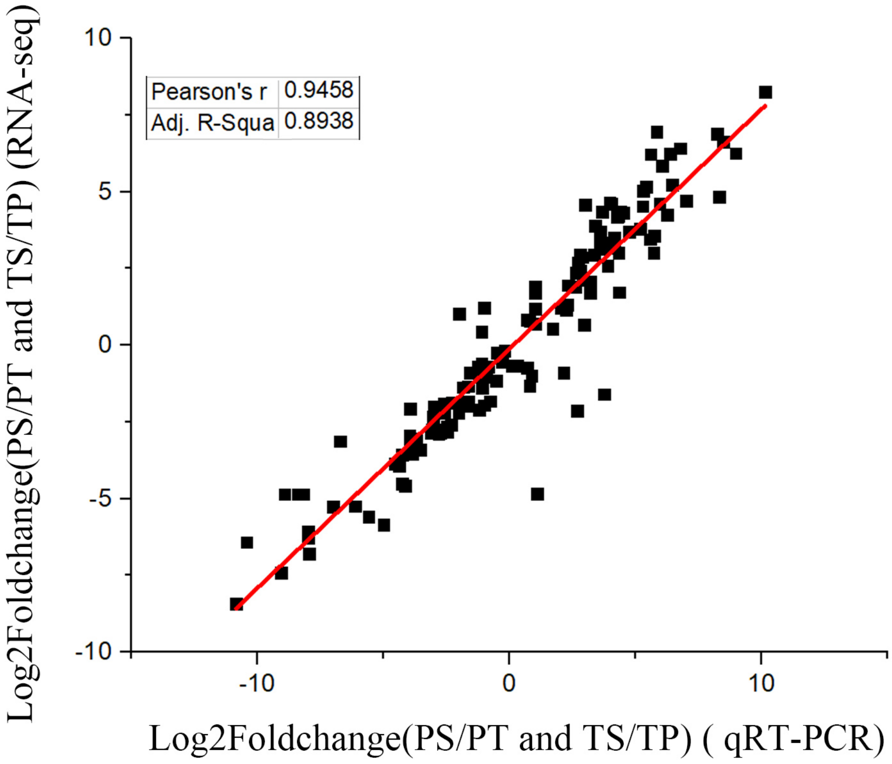

Figure S16. The statistical validation (p <0.05) of 24 genes verified in this study. The linear fitting analysis was performed using the fold change in PS vs PT and TS vs TP of the gene expression levels in qRT-PCR and RNA-seq. Pearson's r was 0.9458.

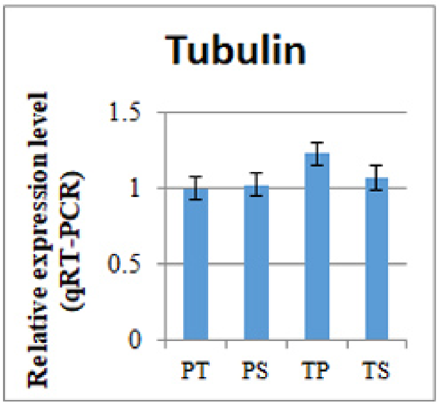

Figure S17. Expression patterns of the tubulin gene by qRT-PCR as negative control gene to compare the expression patterns. The expression levels of this gene were almost the same in the PT, PS, TP and TS pollinations.

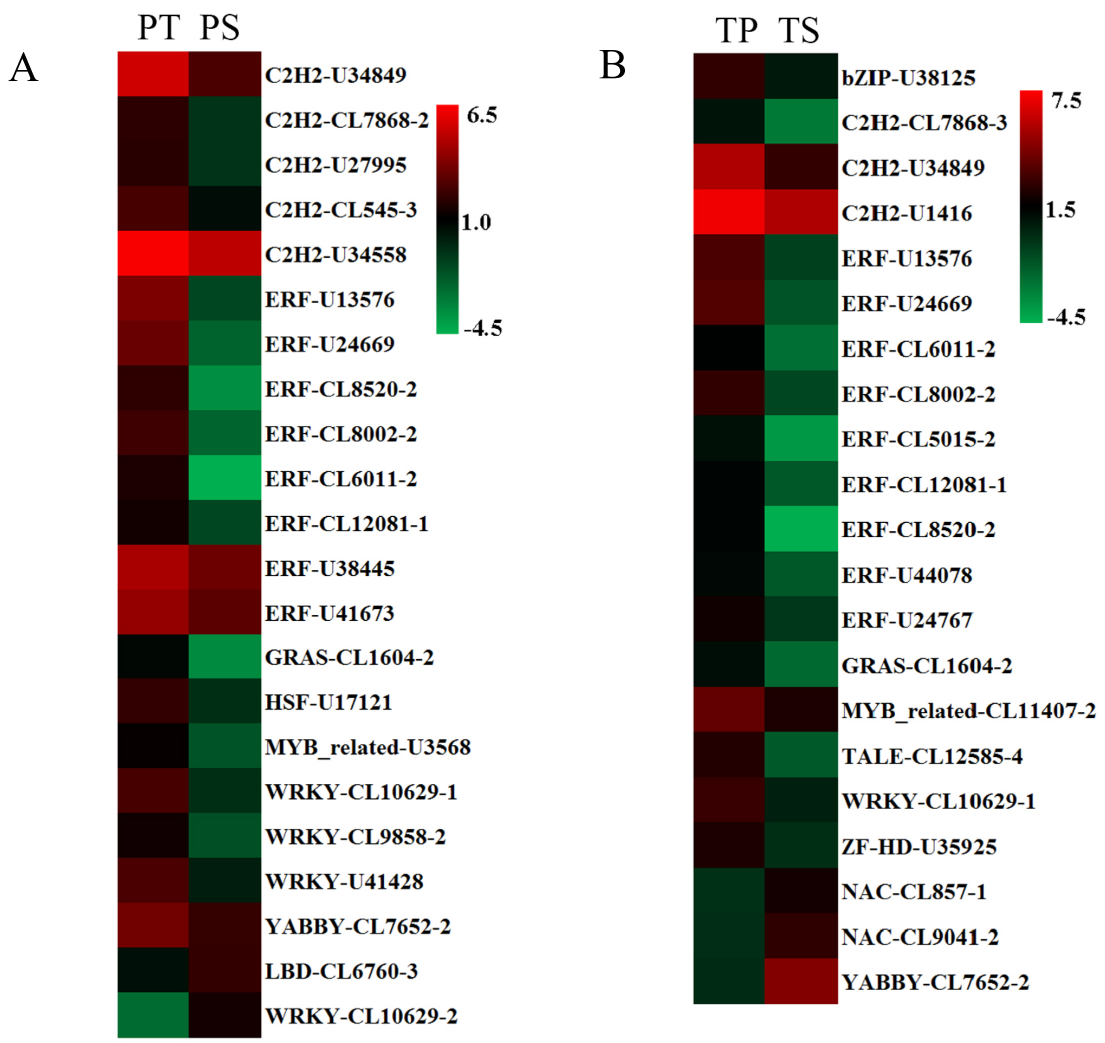

Figure S18. The heat maps of TFs expression. (A) Expression level of the TFs from the DEGs of PS vs PT. (B) Expression level of the TFs from the DEGs of TS vs TP.
