# Supplementary material for: Transcriptomics Investigation into the Mechanisms of Self-Incompatibility between Pin and Thrum Morphs of Primula maximowiczii
Source: Int J Mol Sci. 2018 Jun 22;19(7):1840. doi: 10.3390/ijms19071840 (PMC6073747; doi:10.3390/ijms19071840)
Supplement: Supplementary file 1 [file ijms-19-01840-s001.zip › Supplementary files-2018.06.12/Methods S1 Analysis methods of raw data of RNA-seq.pdf]

Analysis Methods

1 Output Statistics

Raw Sequence Data

Image data output from sequencing machine is transformed by base calling into sequence data, which is called raw data or raw reads and stored in fastq format.

```
@FC61FL8AAXX:1:17:1012:19200#GCCAAT/1
CCACTGTCATGTGAACATCACAGAGACATTTCTTGA
+
bbbbbbbbbbbbbbbbbbbbbbbbbbbaaaaaaaaa_
```

The line 1 and 3 are sequences name generated by the sequencing machines; line 2 is sequence; line 4 is quality letters, of which each letter correspond to a base in line 2; we calculate the sequencing quality of each base in line 2 by subtracting 64 from the ASCII value of the letter in line 4 (sequencing quality value). For example, the ASCII value of c is 99, so the corresponding sequencing quality value is 35. Sequencing quality values range from 2 to 35. Follow table shows the brief relationship between sequencing error rate and sequencing quality value. Denote E as sequencing error rate and sQ as sequencing quality value, then we have:

$$sQ = -10\lg E$$

Brief relationship between sequencing error rate and sequencing quality

Sequencing error rate(E)	Sequencing quality	Corresponding character
5%	13	M
1%	20	T
0.1%	30	^
0.01%	40	h

Data process

Raw reads produced from sequencing machines contain dirty reads which contain adapters, unknown or low quality bases. These data will negatively affect following bioinformatics analysis.

Therefore, dirty raw reads are discarded:

1. Remove reads with adaptors
2. Remove reads with unknown nucleotides larger than 5%
3. Remove low quality reads (The rate of reads which quality value <= 10 is more than 20%)
4. Get the clean reads

Software	Version	Web Site	Parameters
filter_fq	Internal software	-	-

Clean Reads

The following analysis is based on clean reads, which are generated by filtering raw reads.

2 Assembly

Transcriptome de novo assembly is carried out with short reads assembling program - Trinity. Trinity combines three independent software modules: Inchworm, Chrysalis, and Butterfly, applied sequentially to process large volumes of RNA-seq reads. Trinity partitions the sequence data into many individual de Bruijn graphs, each representing the transcriptional complexity at at a given gene or locus, and then processes each graph independently to extract full-length splicing isoforms and to tease apart transcripts derived from paralogous genes. Briefly, the process works like so:

Inchworm Assembles the RNA-seq data into the unique sequences of transcripts, often generating full-length transcripts for a dominant isoform, but then reports just the unique portions of alternatively spliced transcripts.

Chrysalis Clusters the Inchworm Contigs into clusters and constructs complete de Bruijn graphs for each cluster. Each cluster represents the full transcriptonal complexity for a given gene (or sets of genes that share sequences in common). Chrysalis then partitions the full read set among these disjoint graphs.

Butterfly Then processes the individual graphs in parallel, tracing the paths that reads and pairs of reads take within the graph, ultimately reporting full-length transcripts for alternatively spliced isoforms, and teasing apart transcripts that corresponds to paralogous genes.

The result sequences of trinity is called Unigenes. When multiple samples from a same species are sequenced, Unigenes from each sample's assembly can be taken into further process of sequence splicing and redundancy removing with sequence clustering software to acquire non-redundant Unigenes as long as possible. Then do gene family clustering, the Unigenes will be divided to two class. One is clusters, which the prefix is CL and the cluster id is behind. In one cluster, there are several Unigenes which similarity between them is more than 70%. And the other are singletons, which the prefix is Unigene.

In the final step, blastx alignment (evalue < 0.00001) between Unigenes and protein databases like NR, Swiss-Prot, KEGG and COG is performed, and the best aligning results are used to decide sequence direction of Unigenes. If results of different databases conflict with each other, a priority order of NR, Swiss-Prot, KEGG and COG should be followed when deciding sequence direction of Unigenes. When a Unigene happens to be unaligned to non of the above databases, a software named ESTScan will be introduced to decide its sequence direction. For Unigenes with sequence directions, we provide their sequences from 5' end to 3' end; for those without any direction we provide their sequences from assembly software.

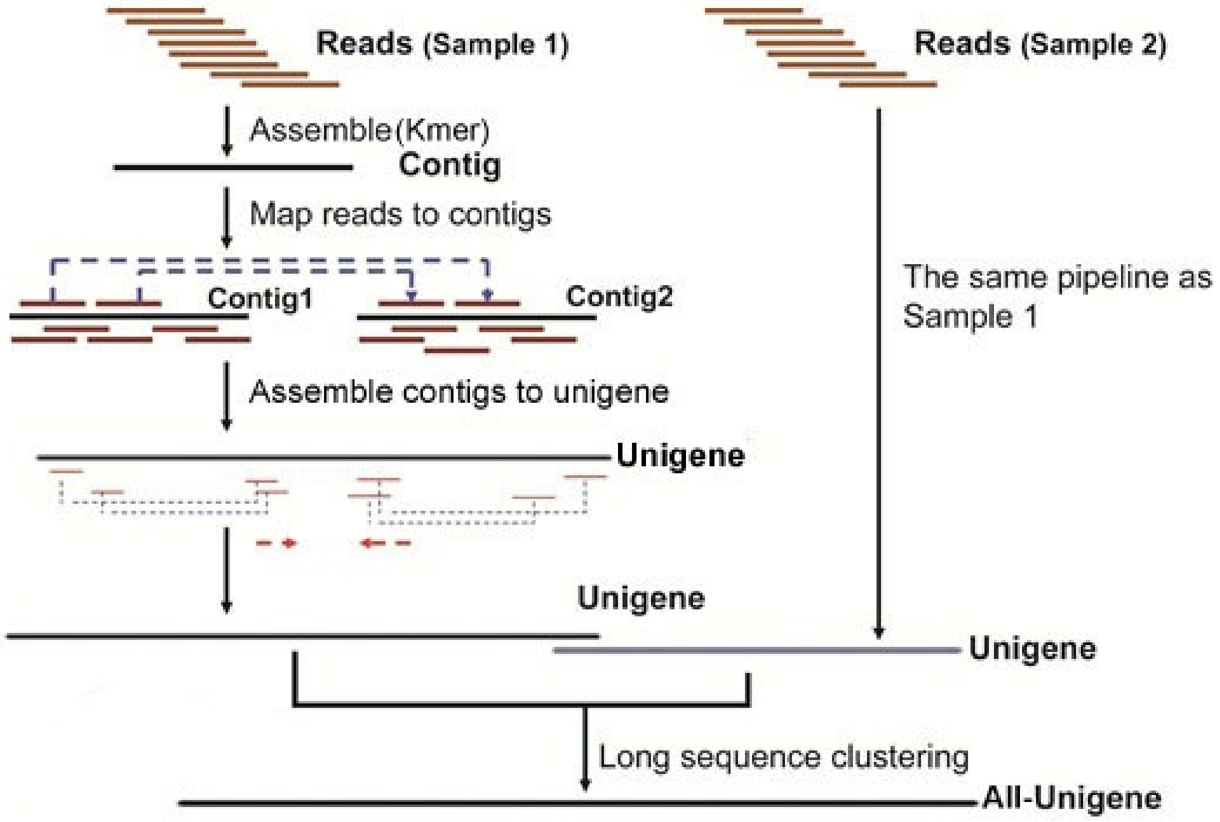

* Assembly process

Software	Version	Web Site	Parameters
Trinity	release-20130225	http://trinityrnaseq.sourceforge.net/	--seqType fq --min_contig_length 100<br>--min_glue 3 --group_pairs_distance 250<br>--path_reinforcement_distance 85 --min_kmer_cov 3
TGICL	v2.1	http://sourceforge.net/projects/tgicl/files/tgicl%20v2.1/	-l 40 -c 10 -v 20
Phrap	Release 23.0	http://www.phrap.org/	-repeat_stringency 0.95 -minmatch 35 -minscore 35

Reference:

[1] Grabherr MG, Haas BJ, et al. Full-length transcriptome assembly from RNA-Seq data without a reference genome. Nature Biotechnology. 2011, 29(7):644-52.

[2] Iseli C, Jongeneel CV, et al. ESTScan: a program for detecting, evaluating, and reconstructing potential coding regions in EST sequences. Proc Int Conf Intell Syst Mol Biol. 1999:138-48.

3 Unigene Function Annotation

KEGG database contains systematic analysis of inner-cell metabolic pathways and functions of gene products. It helps studying complicated biological behaviors of genes. With KEGG annotation we can get Pathway annotation of Unigenes.

COG is a database where orthologous gene products are classified. Every protein in COG is assumed to to evolve from an ancestor protein, and the whole database is built on coding proteins with complete genome as well as system evolution relationships of bacteria, algae and eukaryotic creatures. Unigenes are aligned to COG database to predict and classify possible functions of Unigenes.

NT is non-redundant NCBI nucleotide database, with entries from all traditional divisions of GenBank, EMBL, and DDBJ excluding bulk divisions (gss, sts, pat, est, and htg divisions. wgs entries are also excluded.

Software	Version	Web Site	Database	Release	Parameters
BLAST	v2.2.26+x64-linux	http://blast.ncbi.nlm.nih.gov/Blast.cgi	NT	release-20130408	-F F -e 1e-5 -p blastn
			NR	release-20130408	-F F -e 1e-5 -p blastx
			KEGG	Release 63.0	
			Swiss-Prot	release-2013_03	
			COG	release-20090331	

4 Unigene GO Classification

We can get GO functional annotation with nr annotation. Gene Ontology (GO) is an international standardized gene functional classification system which offers a dynamic-updated controlled vocabulary and a strictly defined concept to comprehensively describe properties of genes and their products in any organism. GO has three ontologies: molecular function, cellular component and biological process. The basic unit of GO is GO-term. Every GO-term belongs to a type of ontology.

With nr annotation, we use Blast2GO program to get GO annotation of Unigenes. Blast2GO has been cited by other articles for more than 150 times and is a widely recognized GO annotation software. After getting GO annotation for every Unigene, we use WEGO software to do GO functional classification for all Unigenes and to understand the distribution of gene functions of the species from the macro level.

Software	Version	Web Site	Database	Release	Parameters
Blast2GO	v2.5.0	http://www.blast2go.com/b2ghome	GO	release 2012-08-01	Default

Reference:

[1] Conesa A, Götz S, et al. Blast2GO: a universal tool for annotation, visualization and analysis in functional genomics research. Bioinformatics.2005, 21(18): 3674-6.

[2] Ye J, Fang L, et al. WEGO: a web tool for plotting GO annotations. Nucleic Acids Res .2006,34(Web Server issue): W293-7.

5 Unigene Metabolic Pathway Analysis

KEGG is a database that is able to anaylze gene product during metabolism process and related gene function in the cellular processes. With the help of KEGG database, we can further study genes’ biological complex behaviors, and by KEGG annotation we can get pathway annotation for Unigenes.

Software	Version	Web Site	Database	Release	Parameters
Path_finder	Internal version	http://www.genome.jp/	KEGG	Release 63.0	Default

Referece:

[1] Kanehisa M, Araki M, et al. KEGG for linking genomes to life and the environment. Nucleic Acids Res. 2008, 36(Database issue): D480-4.

6 Protein Coding Region Prediction(CDS)

Unigenes are firstly aligned by blastx (evalue<0.00001) to protein databases in the priority order of NR, Swiss-Prot, KEGG and COG. That is, we first align Unigenes to NR, then Swiss-prot, then KEGG, and finally COG. Unigenes aligned to a higher priority database will not be aligned to lower priority database.The alignments end when all alignments are finished. Proteins with highest ranks in blast results are taken to decide the coding region sequences of Unigenes, then the coding region sequences are translated into amino sequences with the standard codon table. So both the nucleotide sequences (5'->3') and amino sequences of the Unigene coding region are acquired. Unigenes that cannot be aligned to any database are scanned by ESTScan, producing nucleotide sequence (5’->3’) direction and amino sequence of the predicted coding region.

Using BLAST to analysis CDS on Windows

Download

1. 32 bit: ftp://ftp.ncbi.nlm.nih.gov/blast/executables/release/LATEST/blast-x.x.x-ia32-win32.exe
2. 64 bit: ftp://ftp.ncbi.nlm.nih.gov/blast/executables/release/LATEST/blast-x.x.x-x64-win64.exe

Download appropriate program for your operating system. We provide 32 bit Windows version blast-2.2.23-ia32-win32.exe in our package.

Installation

Take win32 version as an example

1. Double click on blast-2.2.23-ia32-win32.exe, three folders named bin, data and doc will be extracted to current path ("C:\blast" for example).
2. Add "bin" to system path
   Right click on "My Computer" -> Property -> Advanced -> Environment Variables -> double click on "Path" -> add "bin" path in step 1 and separate it from previous items with ";" (";C:\blast\bin"), press "OK" button and reboot your system.
3. If you skipped step 2, you need input full path of the program when you use it.

Run BLAST

1. Format database
   - For nucleotide sequences All-Unigene.blast.cds.fa formatdb -i All-Unigene.blast.cds.fa -p F Drag All-Unigene.blast.cds.fa onto formatN.bat will do the same.
   - For protein sequences All-Unigene.blast.protein.fa formatdb -i All-Unigene.blast.protein.fa -p T Drag All-Unigene.blast.protein.fa onto formatP.bat will do the same.
2. 2.Run blast
   For query nucleotide sequence file seq.fa
   - ◦Seach target nucleotide database All-Unigene.blast.cds.fa using blastn with e-value cutoff 1e-5 and save results in tab-delimited text file named seq.blastn.m8
     blastall -p blastn -i seq.fa -d All-Unigene.blast.cds.fa -e 1e-5 -m 8 -o seq.blastn.m8 Drag seq.fa onto runBlastn.bat can start a command prompt for selecting or inputting one database and then run blastn .
   - Seach target protein database All-Unigene.blast.protein.fa using blastn with e-value cutoff 1e-5 and save results in tab-delimited text file named seq.blastx.m8

blastall -p blastx -i seq.fa -d All-Unigene.blast.protein.fa -e 1e-5 -m 8 -o seq.blastx.m8

Drag seq.fa onto runBlastx.bat can start a command prompt for selecting or inputting one database and then run blastx.

Note

Sequence file used as a database, the path can contain blank spaces, but the file name can not contain blank spaces (not supported by BLAST).

Software	Version	Web Site	Parameters
ESTScan	v3.0.2	http://www.ch.embnet.org/software/ESTScan2.html	Default

Reference:

[1] Iseli C, Jongeneel CV, et al. ESTScan: a program for detecting, evaluating, and reconstructing potential coding regions in EST sequences. Proc Int Conf Intell Syst Mol Biol. 1999,138-48.
